# Effectiveness of food supplements in increasing fat-free tissue accretion in children with moderate acute malnutrition: A randomised 2 × 2 × 3 factorial trial in Burkina Faso

**DOI:** 10.1371/journal.pmed.1002387

**Published:** 2017-09-11

**Authors:** Christian Fabiansen, Charles W. Yaméogo, Ann-Sophie Iuel-Brockdorf, Bernardette Cichon, Maren J. H. Rytter, Anura Kurpad, Jonathan C. Wells, Christian Ritz, Per Ashorn, Suzanne Filteau, André Briend, Susan Shepherd, Vibeke B. Christensen, Kim F. Michaelsen, Henrik Friis

**Affiliations:** 1 Department of Nutrition, Exercise and Sports, University of Copenhagen, Copenhagen, Denmark; 2 Médecins Sans Frontières–Denmark, Copenhagen, Denmark; 3 Département Biomédical et Santé Publique Institut de Recherche en Sciences de la Santé, Ouagadougou, Burkina Faso; 4 Division of Nutrition, St John’s Research Institute, Bangalore, India; 5 Childhood Nutrition Research Centre, UCL Great Ormond Street Institute of Child Health, London, United Kingdom; 6 Center for Child Health Research, University of Tampere School of Medicine and Tampere University Hospital, Tampere, Finland; 7 Faculty of Epidemiology and Population Health, London School of Hygiene & Tropical Medicine, London, United Kingdom; 8 Alliance for International Medical Action, Dakar, Senegal; 9 Department of Paediatrics, Righospitalet, Copenhagen, Denmark; Makerere University Medical School, UGANDA

## Abstract

**Background:**

Children with moderate acute malnutrition (MAM) are treated with lipid-based nutrient supplement (LNS) or corn-soy blend (CSB). We assessed the effectiveness of (a) matrix, i.e., LNS or CSB, (b) soy quality, i.e., soy isolate (SI) or dehulled soy (DS), and (c) percentage of total protein from dry skimmed milk, i.e., 0%, 20%, or 50%, in increasing fat-free tissue accretion.

**Methods and findings:**

Between September 9, 2013, and August 29, 2014, a randomised 2 × 2 × 3 factorial trial recruited 6- to 23-month-old children with MAM in Burkina Faso. The intervention comprised 12 weeks of food supplementation providing 500 kcal/day as LNS or CSB, each containing SI or DS, and 0%, 20%, or 50% of protein from milk. Fat-free mass (FFM) was assessed by deuterium dilution technique. By dividing FFM by length squared, the primary outcome was expressed independent of length as FFM index (FFMI) accretion over 12 weeks. Other outcomes comprised recovery rate and additional anthropometric measures. Of 1,609 children, 4 died, 61 were lost to follow-up, and 119 were transferred out due to supplementation being switched to non-experimental products. No children developed allergic reaction. At inclusion, 95% were breastfed, mean (SD) weight was 6.91 kg (0.93), with 83.5% (5.5) FFM. In the whole cohort, weight increased 0.90 kg (95% CI 0.88, 0.93; *p <* 0.01) comprising 93.5% (95% CI 89.5, 97.3) FFM. As compared to children who received CSB, FFMI accretion was increased by 0.083 kg/m^2^ (95% CI 0.003, 0.163; *p* = 0.042) in those who received LNS. In contrast, SI did not increase FFMI compared to DS (mean difference 0.038 kg/m^2^; 95% CI −0.041, 0.118; *p* = 0.35), irrespective of matrix. Having 20% milk protein was associated with 0.097 kg/m^2^ (95% CI −0.002, 0.196) greater FFMI accretion than having 0% milk protein, although this difference was not significant (*p* = 0.055), and there was no effect of 50% milk protein (0.049 kg/m^2^; 95% CI −0.047, 0.146; *p* = 0.32). There was no effect modification by season, admission criteria, or baseline FFMI, stunting, inflammation, or breastfeeding (*p >* 0.05). LNS compared to CSB resulted in 128 g (95% CI 67, 190; *p <* 0.01) greater weight gain if both contained SI, but there was no difference between LNS and CSB if both contained DS (mean difference 22 g; 95% CI −40, 84; *p* = 0.49) (interaction *p* = 0.017). Accordingly, SI compared to DS increased weight by 89 g (95% CI 27, 150; *p* = 0.005) when combined with LNS, but not when combined with CSB. A limitation of this and other food supplementation trials is that it is not possible to collect reliable data on individual adherence.

**Conclusions:**

Based on this study, children with MAM mainly gain fat-free tissue when rehabilitated. Nevertheless, LNS yields more fat-free tissue and higher recovery rates than CSB. Moreover, current LNSs with DS may be improved by shifting to SI. The role of milk relative to soy merits further research.

**Trial registration:**

ISRCTN registry ISRCTN42569496

## Abstract 2

Malnourished children accrue more fat free tissue with lipid-based food supplements compared to corn-soy blend ones, as revealed in a trial in Burkina Faso led by Christian Fabiansen

## Author summary

**Why was this study done?:**

- Moderate acute malnutrition (MAM) is widespread among children in low-income countries and is a risk factor for morbidity, severe acute malnutrition, and death.
- WHO recommends more research on food supplements for children with MAM. Currently, there is no consensus on either the effectiveness of lipid-nutrient supplement (LNS) compared to corn-soy blend (CSB), or on the role of key factors like milk and soy.
- There is concern that children receiving LNS might accumulate too much fat tissue.

**What did the researchers do and find?:**

- In a large randomised trial of 1,609 children with MAM, conducted in a humanitarian setting in Burkina Faso, we investigated the effectiveness of key factors in supplemental foods, namely (a) the matrix (LNS versus CSB), (b) the soy quality (soy isolate versus dehulled soy), and (c) the level of dry skimmed milk.
- We evaluated body composition to assess the quality of weight gain, and used fat-free mass tissue accretion as our primary outcome.
- Fat-free tissue accretion as well as recovery from MAM were both higher when using LNS compared to CSB.
- Children given LNS did not put on excessive fat.

**What do these findings mean?:**

- Our findings support a wider use of LNS in the treatment of children with MAM. A switch to LNS would lead to greater gain of fat-free tissue and recovery, and would benefit millions of children.

## Introduction

Moderate acute malnutrition (MAM), defined as moderate wasting or low mid-upper arm circumference (MUAC), is widespread among children in low-income countries. While both prevalence and incidence are unknown, moderate wasting alone affects 33 million children at any time [1]. Children with MAM are at risk of morbidity and mortality from infectious diseases or progression to severe acute malnutrition (SAM), while those who recover may be at risk of relapse, impaired physical and cognitive development, and later non-communicable diseases [2,3].

There is limited evidence to inform recommendations on the composition of supplementary foods to treat children with MAM, and the World Health Organization (WHO) recommends research to develop cost-effective products [4]. Supplementary foods for malnourished children are based on a matrix of either corn-soy blend (CSB) or lipid-based nutrient supplement (LNS) [5]. There are substantial differences between the 2 product types, not only in nutritional composition, but also in cost, how they are consumed, and the logistics needed for delivery. A key source of protein in CSB is soy, often dehulled soy (DS), which contains higher levels of anti-nutrients (compounds impairing absorption of minerals) compared to more expensive soy isolate (SI) [6]. The first LNS product was developed to treat SAM and was based on the nutritional composition of the therapeutic milk F-100, containing high amounts of dairy products and no soy [7]. This product type is also referred to as ready-to-use therapeutic food (RUTF). More recently, a range of LNS products, some containing soy, has been developed for MAM. The inclusion of dry skimmed milk (DSM) in supplements improves the amino acid profile, provides minerals with high bioavailability, and reduces the content of anti-nutrients when it replaces vegetable protein sources, but also increases costs [8,9].

Trials assessing the effects of supplements typically use weight gain or nutritional recovery as the primary outcome, and do not consider body composition. While nutritional recovery, i.e., increase in weight-for-height *z*-score (WHZ) or MUAC above specific cutoffs, is an established programmatic outcome, the accompanying health benefits are not well known. Inadequate accretion of fat-free tissue, i.e., muscle, organs, and bone, impairs body function and health [10]. The weight of fat-free tissue, i.e., fat-free mass (FFM), can be measured under field conditions using the deuterium dilution technique and can be expressed independent of length as FFM index (FFMI) by dividing by length in meters squared.

We aimed to investigate the effectiveness of matrix, soy quality, and percentage of total protein from dry skimmed milk in the treatment of MAM in increasing FFMI accretion.

## Methods

### Ethics

The trial was approved by the Ethics Committee for Health Research in Burkina Faso (2012-8-059), and consultative approval was obtained from the Danish National Committee on Biomedical Research Ethics (1208204). The trial was registered in the ISRCTN registry (ISRCTN42569496). Information and consent forms were translated into the local language (Moré) and back-translated to ensure accuracy. Caregivers gave verbal and written consent (signature or fingerprint) prior to enrolment.

### Study design

**Fig 1:**
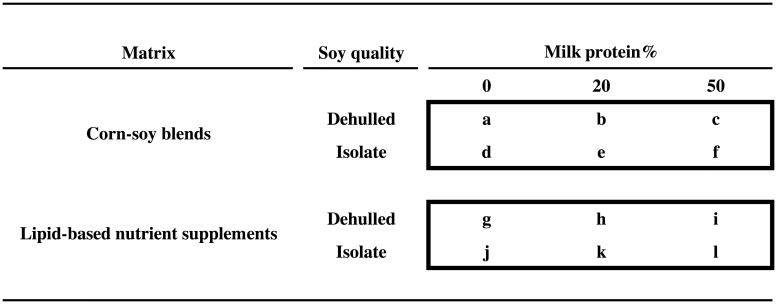
Experimental food supplements. The 2 × 2 × 3 factorial design, showing the 12 experimental food supplements based on corn-soy blends or lipid-based nutrient supplements, with either dehulled soy or soy isolate, and with 0%, 20%, or 50% of total protein from milk. Two supplements correspond to currently used products: “a” (CSB+) and “b” (CSB++). Product “i” is similar to Plumpy’Sup, containing dehulled soy but with dry skimmed milk instead of whey.

We conducted a randomised 2 × 2 × 3 factorial trial (Treatfood) assessing the effects of the matrix (LNS versus CSB), soy quality (SI versus DS), and percentage of protein from DSM (20% and 50% versus 0%), resulting in 12 different supplements, some of which correspond to existing products (Fig 1). Supplements were given for 12 weeks. The trial was double-blinded with respect to soy quality and milk content, but not matrix.

### Participants

This trial was conducted in the Province du Passoré in the northern region of Burkina Faso, in an area qualifying for supplementary feeding programmes based on the prevalence of acute malnutrition [11]. The prevalence of moderate wasting was 9% and of severe wasting and/or oedema was 1.4% [12]. A 3- to 5-month-long ‘lean’ season usually starts in June [13]. The prevalence of HIV infection among women 15–49 years of age was 0.8% [14]. The family diet was cereal-based, and consumption of cow’s milk and other animal-source foods was rare.

Study sites were constructed at 5 governmental health centres (Gomponsom, Latoden, Bagaré, Bokin, and Samba) and staffed by the non-governmental organisation Alliance for International Medical Action (ALIMA, Senegal). The study catchment area was defined by a maximum distance of 23 km from a study site, and villages assumed to be inaccessible during the rainy season were excluded. Population size in the study catchment area was estimated at 258,000.

Children were screened for MAM in villages by community health workers (CHWs) or by designated mobile screening teams. Furthermore, children could be referred to a study site from a health centre, or could present at a site on caregiver’s initiative. Children were recruited if (a) they were resident in the catchment area at the time of inclusion, (b) a diagnosis of MAM was confirmed, i.e., MUAC ≥ 115 mm and < 125 mm and/or WHZ ≥ –3 and < –2, and (c) they were age 6–23 months, based on a health card or a locally adapted events calendar. Children were not included if they were (a) treated for SAM (WHZ < −3 or MUAC < 115 mm or oedema) or hospitalised within the past 2 months, (b) already in a nutritional programme, or (c) requiring hospitalisation, e.g., haemoglobin < 50 g/l. Children with a severe disability, limiting the feasibility of investigations, or with suspected allergy to milk, peanuts, CSB, or LNS were also excluded.

To minimise mixing or sharing of experimental supplements, only the first child with MAM identified from a given household was included in the trial. Siblings with MAM or twins discordant for MAM status received the same supplement but were not included in the trial.

### Randomisation

Children were individually assigned at random to intervention groups. Supplements were designated by a 1-letter code by the manufacturer, and a code-key was kept in a sealed envelope in a safe until completion of data analysis. Random sequences, in blocks of 12 or 24 and stratified by site, were created by a person not involved in the trial using Randomization.com (http://www.randomization.com). The supplements were packed in individual boxes containing a full 12-week treatment for 1 participant (either 6 bags of CSB or 84 sachets of LNS). During production, each box and bag/sachet of supplement was labelled with a 12-letter sequence containing the relevant 1-letter code in a fixed position and the 11 remaining letters in random order. In Burkina Faso, 1 individual not involved in recruitment and data collection was aware of the random sequences and the 1-letter code. This individual relabelled boxes and supplements with individual study IDs, and all later handling of supplements at the 5 study sites was based on study ID alone. At enrolment, children were given a study ID by staff without access to the random sequences or supplements.

### Nutritional intervention

All experimental supplements complied with WHO’s technical note on supplementary foods for the management of MAM [4]. All supplements contained 500 kcal per daily serving (120 g of CSB or 92 g of LNS). The fat content was 11.4–11.7 g per daily serving in CSBs and 31.4–32.1 g per daily serving in LNSs. The protein content was 15.9–16.8 g in CSBs and 12.5–13.5 g in LNSs. All supplements had a protein digestibility—corrected amino acid score (PDCAAS) above 70%, as recommended by WHO [4]. The shelf life for the CSBs was 9 months, and for the LNSs was 2 years. The ingredients in the 12 experimental supplements are shown in Table 1. Soy and maltodextrin replaced milk in milk-free products. More maltodextrin was added when SI replaced DS.

**Table 1:** Supplement composition.

Ingredient	Supplement											
	a	b	c	d	e	f	g	h	i	j	k	l
Corn	55.0	55.0	55.0	55.0	55.0	55.0						
Peanut							27.0	27.0	27.0	27.0	27.0	27.0
Sugar	9.0	9.0	9.0	9.0	9.0	9.0	27.0	27.0	27.0	27.0	27.0	27.0
Maltodextrin	5.6	3.7	0.3	12.55	8.4	2.5	7.8	5.55	1.9	13.47	9.05	2.34
Skimmed milk powder		8.0	20.0		8.0	20.0		8.0	20.0		8.0	20.0
Vegetable fat	7.9	7.8	7.8	8.0	7.9	7.7	18.0	18.0	18.0	18.0	18.0	18.0
Soy protein isolate				11.4	8.1	3.2				8.6	5.5	0.9
Soy flour	19.7	14.0	5.6				15.0	9.5	1.5			
Magnesium citrate	0.5	0.5	0.6	0.85	0.8	0.6	0.2	0.2	0.25	0.33	0.3	0.26
Calcium phosphate	1.4	1.1	0.8	1.5	1.3	0.9	1.95	1.7	1.3	2.1	1.8	1.4
Potassium chloride	0.5	0.5	0.5	1.3	1.1	0.7	0.9	0.9	0.9	1.35	1.2	0.95
Vitamin premix	0.4	0.4	0.4	0.4	0.4	0.4	0.5	0.5	0.5	0.5	0.5	0.5
Emulsifier							1.65	1.65	1.65	1.65	1.65	1.65

Ingredients given as percentage of total weight.

The 6 LNSs had similar packaging and texture, and the 6 CSBs had similar packaging and texture. LNSs were provided in sachets, each containing a daily serving ready for consumption. CSBs were provided in bags of 1.7 kg containing a fortnightly ration (120 g/child/day recommended to be divided in 3 meals, 40 g/meal) to be cooked with water and consumed as a porridge. Supplements were manufactured by GC Rieber Compact (Norway), which was otherwise not involved in the trial design or interpretation of data. The same suppliers for the main raw material, DS, SI, and milk were used for both the LNSs and CSBs. The final experimental supplements were microbiologically tested and approved for food safety by certified labs (Compact India, India; TÜV SÜD, India; Dordogne Labs, France).

### Study visits

Children were scheduled for clinic visits every 2 weeks during the 12-week supplementation period. FFM was assessed at enrolment and after 12 weeks. Clinical status and anthropometry were assessed at every visit, except length, which was measured once a month. Children who missed scheduled visits were visited by a CHW at their homes and encouraged to return for follow-up.

To assess FFM, a dose of 5 g of deuterium oxide (D_2_O) (99.8%; Cambridge Isotope Laboratories, US) diluted in 5 g of bottled water was weighed with 0.01-g precision and given orally after collection of pre-dose saliva samples. Post-dose saliva samples were collected after a 3-hour equilibration period, as established during the pilot study [15]. For each assessment, D_2_O abundance was measured in duplicate in pre- and post-dose saliva samples and in a diluted sample of each child’s dose, using Fourier-transform infrared spectrometry (Agilent Technologies, US) [16] at St. John’s Research Institute, Bangalore, India. At least 60 μl of saliva was required for analysis. Deuterium dilution space was calculated as described previously [17], and converted to total body water (TBW) using a factor of 1.044 to adjust for proton exchange. FFM was calculated as TBW/hydration, using age- and sex-specific hydration coefficients [18]. Fat mass (FM) was calculated as weight minus FFM. Data were cleaned for typographical errors and implausible TBW values, based on the association of TBW with length and cutoffs for FM of <−0.1 (to account for the normal technical variability in deuterium dilution studies) and >2.4 kg. FFM and FM were divided by length in meters squared to derive indices independent of length, i.e., FFM index (FFMI) and FM index (FMI) [19].

Weight was measured in duplicate to the nearest 100 g using electronic scales (Seca model 881 1021659) with double weighing function. Length was measured in duplicate with a wooden height board to the nearest 1 mm (subtracting a correction factor of 0.7 cm for children reaching an age of >24 months during the intervention). WHZ was determined at the study sites using WHO field tables, and this value was used for recruitment. In later analysis, WHZ was recalculated using the package “zscore06” in STATA 12 (StataCorp, US). MUAC was measured in duplicate to the nearest 1 mm, at the midpoint between the olecranon and the acromion process using a standard measuring tape. Triceps skinfold was measured in duplicate using a Harpenden calliper. The mean of the duplicate measurements was used for analysis. Knee-heel length was measured using a digital calliper (Mitutoyo, Germany), mounted with knee and heel caps cast in hard plastic [20]. The instrument has a resolution of 0.01 mm. The result was expressed as the mean of 5 consecutive separate measurements. Anthropometric measurements were undertaken by trained staff, after standardisation sessions.

At enrolment, a research nurse collected data on demographic characteristics, vaccination status, and 2-week retrospective morbidity and medical treatments using a structured questionnaire in the local language. All children received vitamin A supplementation (100,000 IU if 4–8 kg; 200,000 IU if >8 kg) if they had not received any supplements in the previous 6 months, and albendazole (200 mg if 4–8 kg; 400 mg if >8 kg) and vaccinations were administered according to the national schedule at the health centres. Medical treatment was provided based on Integrated Management of Childhood Illness guidelines. Venous blood was collected from the arm and tested for *Plasmodium falciparum* by rapid test (SD Bioline Malaria Ag P.f; Standard Diagnostics, Korea). Serum C-reactive protein was determined using a combined sandwich enzyme-linked immunosorbent assay (VitMin Lab, Germany).

Nutritional recovery was categorised into 3 groups: recovery at 12 weeks (WHZ ≥ −2 and MUAC ≥ 125 mm), MAM (non-response) at 12 weeks, or SAM at any point during the intervention. Children developing SAM were discontinued from experimental supplements, treated with RUTF, and transferred to inpatient treatment if accompanied by medical complications.

Children who did not recover after 12 weeks of supplementation received 2 weeks of RUTF. Children who did not gain weight during this period were examined by research nurses and if necessary referred to hospital. All children who had not recovered from MAM after 2 weeks of RUTF supplementation received it for a further 2 weeks. Children who failed to recover after the total 4 weeks of RUTF supplementation were referred to hospital for a more in-depth clinical examination.

During the trial, due to suspected contamination of 1 batch of experimental supplement, the commercially available LNS Plumpy’Sup (Nutriset, France) was given to some children.

### Outcomes

The primary outcome was FFMI increment measured using the deuterium dilution technique. D_2_O enrichment was determined in a single laboratory, and cleaning of data was blinded. Other outcomes included increments in FFM, FM, FMI, weight, length, knee-heel length, MUAC, triceps skinfold, and nutritional recovery. Serious adverse events were not expected from intake of the food supplements, but were defined as anaphylactic reaction and death. The effects were expressed as LNS compared to CSB, SI compared to DS, and 20% and 50% milk protein compared to 0% milk protein.

### Statistical analyses

To detect a 0.6-SD difference between any 2 combinations of the 3 factors with 80% power and 5% significance, while allowing for 20% loss to follow-up, we aimed to recruit in total 1,608 (= 12 × 134) children.

Data were double-entered in EpiData 3.1 (Denmark). Statistical analyses were carried out using STATA 12. In the absence of an unsupplemented group, baseline values were regressed on age to estimate average age-associated weight and FFM accretion over a period equivalent to the length of the intervention. Descriptive statistics are presented as mean (SD). Mean changes in anthropometry and body composition are given with 95% confidence intervals.

The analysis was based on the intention-to-treat (ITT) principle using available case data. The 2 × 2 × 3 factorial design was analysed using a linear mixed model with site-specific random effects. Model reduction was carried out to reduce the 3-way interaction to 2-way interactions or main effects (using likelihood ratio tests). Specifically, the full model including the 3-way interaction corresponding to the 2 × 2 × 3 factorial design was compared to the model containing only the main effects of the 3 factors; thus, we tested for any kind of interaction in the factorial layout in one go. Pairwise comparisons of means were performed using model-based post hoc *t* tests in the reduced models. The effects of supplementation are presented in terms of estimated means with 95% CIs. All analyses included adjustment for baseline measure of the outcome [21], age, sex, MUAC, WHZ, and month of admission with the exception of unadjusted ITT analyses, which included only baseline measure of outcome and site. A per protocol (PP) analysis, including only children who completed 12 weeks of supplementation with the supplement to which they were originally allocated, was also conducted.

Effect modification was assessed for season, admission criteria, and baseline FFMI, stunting, inflammation, and breastfeeding. Model checking was based on residuals and normal probability plots. Recovery was evaluated by means of chi-squared tests. A significance level of 0.05 was applied.

Compared to the statistical plan outlined in the protocol (S1 Text), we deviated on 2 issues. First, we adjusted FFM for length by dividing it by length in meters squared, i.e., expressing FFM accretion as an index, to be able to adjust for not only baseline length but also changes in length over time. Second, we initially planned to adjust for multiplicity, in case we had to compare 12 groups. However, in the reduced factorial model with pairwise comparisons within factors, no multiplicity adjustments were applied.

## Results

**Fig 2:**
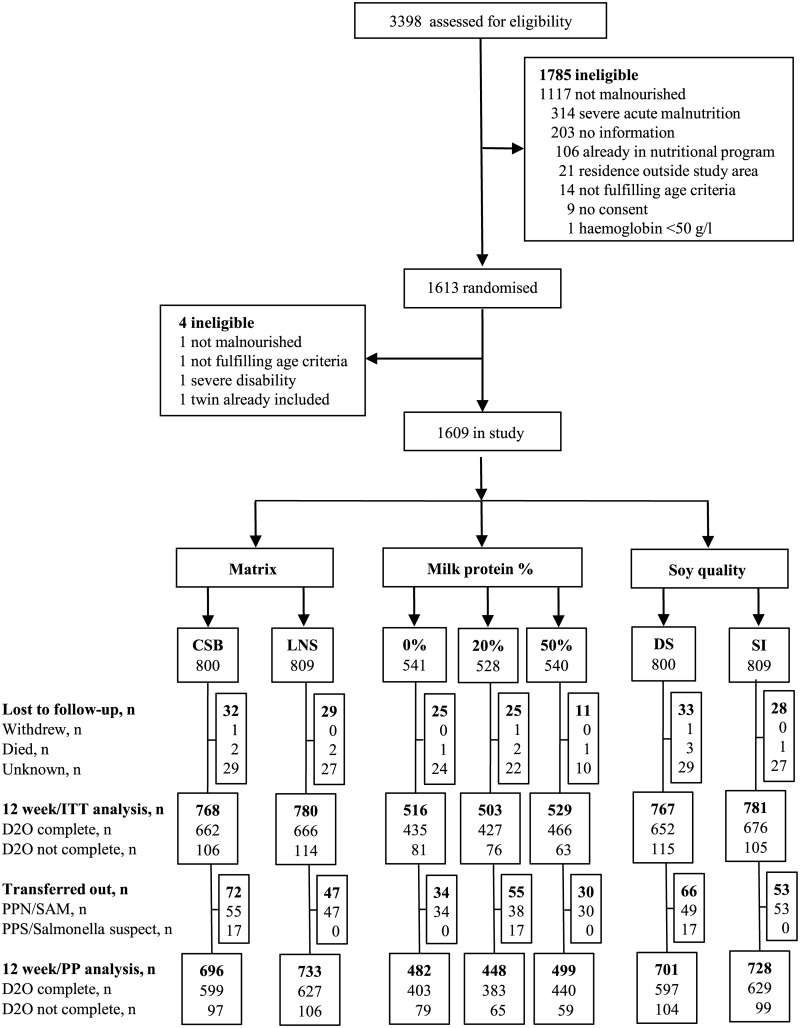
Trial profile. In all, 3,398 children were assessed for eligibility at the 5 study sites, and 1,609 children were randomised and included in the study. CSB, corn-soy blend; D20, deuterium oxide measurement; DS, dehulled soy; ITT, intention-to-treat; LNS, lipid-based nutrient supplement; PP, per protocol; PPS/Salmonella, received Plumpy’Sup due to suspicion of salmonella contamination; PPN/SAM, received Plumpy’Nut due to severe acute malnutrition; SI, soy isolate.

From September 9, 2013, to August 29, 2014, we assessed 3,398 children, of whom 1,967 (58%) had MAM. Of these, 1,613 (82%) children were randomised, but 4 excluded as ineligible. Hence, a total of 1,609 children were randomised according to the 2 × 2 × 3 factorial design (Fig 2).

As shown in Table 2, the randomisation resulted in baseline equivalence between categories of the experimental factors with respect to potential confounders.

**Table 2:** Baseline characteristics by factors.

Characteristic	Matrix		Soy quality		Milk protein percent		
	CSB (*n* = 800)	LNS (*n* = 809)	Dehulled (*n* = 800)	Isolate (*n* = 809)	0% (*n* = 541)	20% (*n* = 528)	50% (*n* = 540)
**Sociodemographic data**							
Age (months)	11 (8–16)	12 (8–16)	11 (8–16)	11 (8–16)	11 (8–16)	11 (8–16)	11 (8–16)
Sex female	444 (56%)	435 (54%)	427 (53%)	452 (56%)	295 (55%)	287 (54%)	297 (55%)
Maternal age (years) (*n* = 1,580)	26 (22–31)	27 (22–31)	27 (22–31)	26 (22–31)	27 (22–31)	26 (22–31)	27 (22–31)
Household size	10 (7–16)	11 (7–16)	11 (8–16)	10 (7–16)	10 (7–15)	10 (7–16)	11 (7–17)
**Anthropometric data**							
Weight-for-height *z*-score	−2.2 (0.5)	−2.2 (0.5)	−2.2 (0.5)	−2.2 (0.5)	−2.2 (0.5)	−2.2 (0.5)	−2.2 (0.5)
Mid-upper arm circumference (mm)	123 (4)	123 (4)	123 (4)	123 (4)	122 (4)	123 (4)	123 (4)
**Admission criteria**							
Weight-for-height *z*-score and mid-upper arm circumference (mm)	404 (51%)	400 (49%)	406 (51%)	398 (49%)	276 (51%)	275 (52%)	253 (47%)
Weight-for-height *z*-score only	171 (21%)	166 (21%)	168 (21%)	169 (21%)	111 (21%)	110 (21%)	116 (22%)
Mid-upper arm circumference (mm) only	225 (28%)	243 (30%)	226 (28%)	242 (30%)	154 (29%)	143 (27%)	171 (32%)
**Paraclinical data**							
Malaria rapid test positive (*n* = 1,601)	324 (41%)	320 (40%)	322 (41%)	322 (40%)	216 (40%)	207 (39%)	221 (41%)
Serum C-reactive protein > 15 mg/l (*n* = 1,564)	148 (19%)	145 (18%)	154 (20%)	139 (18%)	100 (19%)	113 (22%)	80 (15%)
**Breastfed (*n* = 1,607)**	755 (95%)	766 (95%)	755 (95%)	766 (95%)	515 (95%)	493 (93%)	513 (95%)

Data are n (percent of non-missing data) or mean (SD) or median (lower quartile—upper quartile). If data are missing, n is listed.

CSB, corn-soy blend; LNS, lipid-based nutrient supplement.

During the intervention, 61 (3.8%) children were lost to follow-up, leaving 1,548 (96.2%) for ITT analyses. Four children died and no child developed an allergic reaction to supplements. The intervention was discontinued before 12 weeks for 119 children: 17 children were supplemented with Plumpy’Sup due to unconfirmed suspicion of salmonella contamination of their experimental supplement, while 102 children deteriorating into SAM were switched to therapeutic foods. Thus, the PP analyses included 1,429 (88.8%) children.

Baseline weight mean (SD) was 6.91 kg (0.93). Weight at baseline comprised 83.5% (5.5) FFM. We estimated, based on regression of baseline data on age, that weight would increase by 469 g (95% CI 453, 485) and FFM by 434 g (95% CI 416, 453) over 3 months in the absence of supplementation. During the intervention, weight increased 0.90 kg (95% CI 0.88, 0.93), and 93.5% (95% CI 89.6, 97.4) of the weight gain comprised FFM. WHZ increased from −2.22 (0.51) by 0.68 (95% CI 0.65, 0.72), while the height-for-age *z*-score, which was −1.70 (1.1) at baseline, declined by 0.17 (95% CI 0.15, 0.19) (Table 3).

**Table 3:** Changes in body composition and anthropometry during the intervention period.

Outcome	Baseline		Endline		Difference		
	*n*	Mean (SD)	*n*	Mean (SD)	*n*	Mean (SD)	95% CI
**Primary outcome**							
Fat-free mass index (kg/m^2^)	1,489	11.62 (0.87)	1,425	12.41 (0.92)	1,328	0.80 (0.95)	0.74, 0.85
**Other outcomes**							
Fat-free mass (kg)	1,489	5.79 (0.91)	1,425	6.61 (0.98)	1,328	0.85 (0.49)	0.82, 0.87
Fat mass index (kg/m^2^)	1,489	2.30 (0.78)	1,425	2.26 (0.81)	1,328	−0.05 (0.93)	−0.10, −0.002
Fat mass (kg)	1,489	1.13 (0.39)	1,425	1.19 (0.42)	1,328	0.059 (0.48)	0.033, 0.084
Weight (kg)	1,609	6.91 (0.93)	1,548	7.81 (1.05)	1,548	0.90 (0.47)	0.88, 0.93
Length (cm)	1,609	70.4 (5.3)	1,548	72.9 (5.1)	1,548	2.6 (1.0)	2.5, 2.6
Weight-for-height *z*-score	1,609	−2.22 (0.51)	1,548	−1.53 (0.72)	1,548	0.68 (0.71)	0.65, 0.72
Knee-heel length (mm)	1,608	192.2 (18.1)	1,547	203.0 (18.2)	1,546	10.8 (6.4)	10.5, 11.1
Mid-upper arm circumference (mm)	1,609	122.6 (4.0)	1,548	130.1 (6.7)	1,548	7.5 (6.1)	7.2, 7.8
Triceps skinfold (mm)	1,607	6.31 (1.08)	1,548	6.89 (1.26)	1,546	0.57 (1.17)	0.51, 0.63
Height-for-age *z*-score	1,609	−1.70 (1.12)	1,548	−1.86 (1.06)	1,548	−0.17 (0.40)	−0.19, −0.15

LNS increased FFMI accretion by 0.083 kg/m^2^ (95% CI 0.003, 0.163) compared to CSB. In contrast, SI did not increase FFMI (0.038 kg/m^2^; 95% CI −0.041, 0.118) compared to DS, irrespective of matrix. Having 20% milk protein was associated with 0.097 kg/m^2^ (95% CI −0.002, 0.196) greater FFMI accretion than having 0% milk protein, although this difference was not significant (*p* = 0.055), and there was no effect of 50% milk protein (0.049 kg/m^2^; 95% CI −0.047, 0.146) (Table 4).

**Table 4:** Effects of supplementary foods on body composition and anthropometry: Intention-to-treat analysis.

Outcome	Matrix: LNS versus CSB	Soy quality: Isolate versus dehulled	Milk protein percent	
			20% versus 0%	50% versus 0%
**Primary outcome**				
Fat-free mass index (kg/m^2^)	0.083 (0.003, 0.163)	0.038 (−0.041, 0.118)	0.097 (−0.002, 0.196)	0.049 (−0.047, 0.146)
**Other outcomes**				
Fat-free mass (g)	37 (−10, 84)	24 (−23, 71)	41 (−18, 99)	29 (−28, 87)
Fat mass index (kg /m^2^)	0.052 (−0.023, 0.127)	−0.002 (−0.077, 0.073)	−0.085 (−0.178, 0.008)	−0.063 (−0.155, 0.028)
Fat mass (g)	28 (−12, 67)	1 (−38, 41)	−41 (−90, 8)	−30 (−77, 18)
Weight (g)	75 (32, 119)^1^	36 (−8, 79)	9 (−45, 63)	20 (−33, 73)
	With SI: 128 (67, 190)	In LNS: 89 (27, 150)		
	With DS: 22 (−40, 84)	In CSB: −18 (−80, 44)		
Length (cm)	0.03 (−0.06, 0.11)	0.02 (−0.07, 0.10)	0.03 (−0.08, 0.13)	0.07 (−0.03, 0.17)
Weight-for-height *z*-score	0.11 (0.04, 0.17)	0.05 (−0.01, 0.11)	0 (−0.07, 0.08)	0.01 (−0.07, 0.09)
Knee-heel length (mm)	0.4 (−0.2, 1.0)	0 (−0.6, 0.6)	0.6 (−0.2, 1.3)	0.7 (−0.1, 1.4)
Mid-upper arm circumference (mm)	1.0 (0.5, 1.6)	0.3 (−0.2, 0.9)	0.5 (−0.2, 1.1)	0.2 (−0.5, 0.9)
Triceps skinfold (mm)	0.16 (0.06, 0.25)	0.06 (−0.04, 0.16)	−0.05 (−0.17, 0.07)	−0.09 (−0.21, 0.03)

Data are mean difference (95% CI) based on intention-to-treat population. There were no interactions between the 3 factors (all p > 0.11) except for in the case of weight. Linear mixed models adjusted for baseline measure of the outcome, mid-upper arm circumference, weight-for-height z-score, and age, as well as month of admission, sex, and site (random effects).

^1^Interaction between matrix and soy quality (p = 0.017).

DS, dehulled soy; CSB, corn-soy blend; LNS, lipid-based nutrient supplement; SI, soy isolate.

There was no effect modification by season, admission criteria, or baseline FFMI, stunting, inflammation, or breastfeeding (*p >* 0.05). For weight gain, an interaction between matrix and soy quality was found (*p* = 0.017). Hence, the 75 g (95% CI 32, 119) main effect of LNS shown in Table 4 reflects a 128 g (95% CI 67, 190) larger gain when giving LNS compared to CSB if both contained SI, but no difference between LNS and CSB if both contained DS (22 g; 95% CI −40, 84). Similarly, the main 36 g (95% CI −8, 79) effect of SI compared to DS reflects that SI increased weight by 89 g (95% CI 27, 150) compared to DS when combined with LNS, but not when combined with CSB (−18 g; 95% CI −80, 44). LNS compared to CSB had an effect on both MUAC (mean difference 1 mm; 95% CI 0.5, 1.6) and triceps skinfold (mean difference 0.16 mm; 95% CI 0.06, 0.25). None of the factors had effects on length and knee-heel length, although 50% milk protein was associated with a 0.07 cm (95% CI −0.01, 0.14) knee-heel length gain (*p* = 0.07) compared to 0% milk protein.

Overall, the recovery rate was 7% higher for LNS versus CSB (69% versus 62%, *p* = 0.002), and the non-response rate was 6% lower (24% versus 30%, *p* = 0.007); there was no difference in development of SAM (7.3% versus 8.7%, *p* = 0.31). There were no differences between DS and SI with respect to recovery rate (65% versus 66%), non-response rate (28% versus 26%), and risk of SAM (7.6% versus 8.5%) (all *p >* 0.43). Likewise, there were no differences between 0%, 20%, and 50% milk protein in recovery rate (66%, 65%, and 65%), non-response rate (26%, 26%, and 28%), and risk of SAM (8.1%, 8.8%, and 7.2%) (all *p >* 0.65).

Unadjusted analyses and PP analyses showed similar results to ITT analyses (Tables 5 and 6).

**Table 5:** Effects of supplementary foods on body composition and anthropometry: Unadjusted intention-to-treat analysis.

Outcome	Matrix: LNS versus CSB	Soy quality: Isolate versus dehulled	Milk protein percent	
			20% versus 0%	50% versus 0%
**Primary outcome**				
Fat-free mass index (kg/m^2^)	0.078 (−0.009, 0.165)	0.026 (−0.062, 0.113)	0.081 (−0.028, 0.189)	0.056 (−0.050, 0.162)
**Other outcomes**				
Fat-free mass (g)	43 (−8, 94)	18 (−33, 70)	38 (−25, 102)	30 (−32, 92)
Fat mass index (kg/m^2^)	0.052 (−0.028, 0.132)	0.004 (−0.076, 0.085)	−0.084 (−0.184, 0.015)	−0.060 (−0.158, 0.037)
Fat mass (g)	37 (−5, 79)	5 (−37, 47)	−35 (−88, 17)	−28 (−79, 23)
Weight (g)	77 (31, 123)	37 (−9, 83)	1 (−56, 58)	10 (−45, 66)
	With SI: 123 (59, 188)	In LNS: 83 (18, 148)		
	With DS: 30 (−35, 95)	In CSB: −10 (−75, 55)		
Length (cm)	0.03 (−0.06, 0.12)	0.02 (−0.07, 0.11)	0.01 (−0.10, 0.12)	0.07 (−0.03, 0.18)
Weight-for-height *z*-score	0.11 (0.05, 0.18)	0.05 (−0.01, 0.12)	0 (−0.08, 0.08)	0.01 (−0.07, 0.09)
Knee-heel length (mm)	0.55 (−0.07, 1.17)	−0.02 (−0.64, 0.60)	0.49 (−0.28, 1.25)	0.66 (−0.09, 1.42)
Mid-upper arm circumference (mm)	1.2 (0.6, 1.8)	0.3 (−0.3, 0.9)	0.4 (−0.3, 1.1)	0.2 (−0.5, 0.9)
Triceps skinfold (mm)	0.17 (0.07, 0.27)	0.06 (−0.04, 0.16)	−0.05 (−0.18, 0.07)	−0.09 (−0.22, 0.03)

Data are mean difference (95% CI) based on intention-to-treat population. Linear mixed models adjusted for baseline measure of the outcome and site (random effects).

DS, dehulled soy; CSB, corn-soy blend; LNS, lipid-based nutrient supplement; SI, soy isolate.

**Table 6:** Effects of supplementary foods on body composition and anthropometry, per protocol analysis.

Outcome	Matrix: LNS versus CSB	Soy quality: Isolate versus dehulled	Milk protein percent	
			20% versus 0%	50% versus 0%
**Primary outcome**				
Fat-free mass index (kg/m^2^)	0.088 (0.005, 0.171)	0.057 (−0.027, 0.140)	0.106 (0.001, 0.210)	0.054 (−0.046, 0.155)
**Other outcomes**				
Fat-free mass (g)	36 (−14, 85)	35 (−14, 85)	47 (−14, 109)	30 (−29, 90)
Fat mass index (kg/m^2^)	0.077 (−0.001, 0.154)	0.005 (−0.073, 0.083)	−0.101 (−0.198, −0.004)	−0.067 (−0.161, 0.027)
Fat mass (g)	40 (−1, 81)	6 (−35, 47)	−49 (−100, 2)	−29 (−79, 19)
Weight (g)	84 (39, 129)	50 (5, 95)	6 (−50, 62)	22 (−32, 77)
	With SI: 128 (65, 191)	In LNS: 93 (31, 156)		
	With DS: 39 (−26, 103)	In CSB: 4 (−60, 68)		
Length (cm)	0.02 (−0.06, 0.11)	0.03 (−0.06, 0.12)	0.02 (−0.08, 0.13)	0.06 (−0.04, 0.17)
Weight-for-height *z*-score	0.12 (0.06, 0.19)	0.07 (0.01, 0.14)	0 (−0.08, 0.08)	0.01 (−0.07, 0.09)
Knee-heel length (mm)	0.5 (−0.1, 1.2)	0.1 (−0.6, 0.7)	0.6 (−0.2, 1.3)	0.7 (−0.03, 1.49)
Mid-upper arm circumference (mm)	1.2 (0.7, 1.8)	0.5 (−0.1, 1.0)	0.4 (−0.3, 1.1)	0.2 (−0.4, 0.9)
Triceps skinfold (mm)	0.16 (0.06, 0.26)	0.06 (−0.04, 0.16)	−0.06 (−0.19, 0.07)	−0.09 (−0.21, 0.04)

Data are mean difference (95% CI) based on per protocol population.

Linear mixed models adjusted for baseline measure of the outcome, mid-upper arm circumference, weight-for-height z-score and age, as well as month of admission, sex and site.

DS, dehulled soy; CSB, corn-soy blend; LNS, lipid-based nutrient supplement; SI, soy isolate.

## Discussion

There is a paucity of evidence to inform policy on the composition of supplementary foods for children with MAM. Our trial investigated the effectiveness of the key factors matrix, soy quality, and milk protein in food supplements used to treat young Burkinabe children with MAM identified by moderate wasting [22] or low MUAC, which is often used to identify MAM in nutritional programmes [23]. The 12 weeks of supplementation resulted in high gain of FFM across the intervention groups. FFMI was higher in children receiving LNS compared to CSB, and weight gain was greater in children receiving LNS with SI compared to LNS with DS.

Several studies have evaluated the effect of supplementation on weight in children with MAM, but data on body composition are lacking [24–31]. A recent study from Mali that applied the same anthropometric admission criteria as in our study and that also supplemented with 500 kcal/day for 12 weeks reported an overall weight gain similar to our study [29]. The average weight gain during our intervention was about 0.9 kg and predominately comprised FFM. For ethical reasons, we did not include an unsupplemented control group. Nevertheless, based on our baseline data, we estimated that the interventions roughly doubled both weight and FFM accretion in our cohort overall, which is supported by our finding that WHZ increased by 0.68.

Interestingly, despite the diagnosis of MAM, the large proportion of weight accreted as FFM in our cohort is consistent with what would be expected among well-nourished children [32]. All experimental supplements provided the full range of nutrients for synthesis of muscle and organ tissue, which partly explains the high FFM accretion. A larger FFM requires a larger amount of fat to buffer a given period of starvation; hence, accretion of fat is expected to accompany FFM accretion. The benefit of fat was previously shown by low levels of leptin, a hormonal correlate of fat, predicting mortality from SAM [33]. The modest increase in fatness in our study is unlikely to be detrimental.

Older studies of nutritional rehabilitation of children with SAM reported high rates of fat accretion [34,35], and this was replicated in a study of rapid recovery from SAM in Indian children [36]. These findings may partly be explained by diets not being adequately fortified with zinc and other growth nutrients, i.e., nutrients essential to synthesis of fat-free tissue [37,38]. These studies involved very high weight gain velocities, up to 14 times that of a well-nourished child and about 7 times faster than in our study. Conversely, no excess fat in children with SAM rehabilitated with relatively slow rates of weight gain was reported in a recent small study using the deuterium dilution technique to measure FFM [39]. Our study, with longitudinal body composition data in children with MAM, provides more robust evidence that moderate weight gain through nutritional supplementation can prioritise fat-free tissue accretion, with little increase in adiposity.

LNS increased our primary outcome, FFMI, a measure of FFM independent of length. This increase shows that LNS compared to CSB leads to more fat-free tissue, which is likely to mediate beneficial functional outcomes. In addition, LNS had main effects on several other outcomes, increasing WHZ, MUAC, triceps skinfold, and the rate of nutritional recovery. These benefits of LNS could be due to higher bioavailability of growth nutrients, such as zinc and phosphorus, compared to CSB, or a relatively higher intake. Both in a pilot study and in the trial population, we have shown that children allocated LNS consumed a larger proportion of their supplement than did those allocated CSB [40–42].

For weight, the interaction identified between matrix and soy quality reflected that LNS with SI yielded an 89 g (approximately 10% total gain) greater weight gain than did LNS with DS. The soy quality had no effect on weight for CSB. This was unexpected as SI compared to DS contains fewer anti-nutrients, which should lead to better absorption of minerals [6]. Interestingly, we previously found a similar strong interaction between matrix and soy quality with respect to a supplement appreciation score, which indicated that children liked DS better than SI in CSB, but not in LNS [41].

There were no overall effects of milk, probably because 95% of the children were breastfed. However, the point estimate of the effect on FFMI of having 20% compared to no milk protein was similar to that of LNS versus CSB, although not significant.

Our study was not set up to investigate cost-effectiveness, which is complicated by the fact that the prices of ingredients fluctuate considerably. Based on international market prices (October 2016) and calculated per gram protein, the cost of SI is about 80% of the price of DSM, and the cost of DS is 33% of the price of DSM. Moreover, a crude estimate based on information from UNICEF (October 2016) is that LNS is about twice as expensive as CSB per daily ration given [43]. However, such a comparison does not take into account the faster recovery with LNS as well as other associated costs. For example, CSB takes time to prepare, has shorter shelf life, and is bulkier, with implications for transport and storage. Detailed cost-effectiveness studies are needed to inform policy. Our findings support a wider use of LNS, which may also further strengthen future initiatives towards an integrated approach to the management of MAM and SAM with the use of a single product, which may well increase the effectiveness of programmes and reduce their overall cost per child [44].

Our trial has several strengths and limitations. One strength is that the factorial design allowed us to assess the effect of key generic factors in supplements, rather than comparing products differing with respect to multiple factors. It also allowed us to assess possible interactions, and, if none, to have more power to estimate main effects. For example, this enabled us to have power to detect a main effect of LNS compared to CSB on FFMI, and to identify interactions between matrix and soy quality with respect to weight gain. Yet, a limitation inherent to food intervention trials is that if an ingredient containing macronutrients is added to a product, then the content of other ingredients must be adjusted to keep the amount of macronutrients and energy per dose constant, which complicates interpretation. In our trial, for example, when DS is replaced by SI, then the amount of maltodextrin is increased. Similarly, when milk is added, then the amount of soy as well as maltodextrin is reduced. So, in effect, when we see a strong, although not significant, association between 20% milk and FFMI, then it is compared to no milk, but more soy and maltodextrin. Furthermore, any difference between milk and soy may not be due to their protein quality alone, but also to effects of accompanying nutrients or anti-nutrients. For example, milk was included as skimmed milk, which also contains lactose and highly bioavailable minerals. Future research on the relative merit of milk and soy proteins should use pure protein.

Another strength is that we assessed body composition, since a poor-quality diet will lead to accretion of fat at the expense of fat-free tissue, which is essential for functional outcomes. Furthermore, we measured FFM by the state-of-the-art deuterium dilution technique. For our primary outcome, we expressed FFM as FFMI to disentangle FFM accretion from length increase and to increase precision. The body composition data also allowed us to conclude that concern over whether the high content of fat in LNS may lead to high fat accretion is unjustified. Although we did find increased skinfold thickness in children given LNS, the level of fat accretion observed is unlikely to be harmful.

One limitation of our study is that the effect of a nutritional intervention depends on background nutritional status and intake, and the lack of this information limits generalisability. Furthermore, it is not feasible to collect detailed data at the individual level on intake of a supplement in a large trial in children. Instead, we conducted sub-studies on acceptability and feeding behaviour in relation to the supplements, to facilitate interpretation of the findings from the trial. However, our study was an effectiveness study, and the use of supplements in the trial probably came close to their actual use in a nutritional programme. Thus, irrespective of the underlying mechanisms, the differences we identified between the factors tested, particularly for in our primary outcome FFMI, are highly relevant for policy.

In conclusion, children with MAM when nutritionally rehabilitated with supplements providing the full range of nutrients put on predominately fat-free tissue. Nevertheless, LNS yielded more fat-free tissue and higher recovery rates than CSB. Moreover, current LNS products with DS may be improved by shifting to SI. The role of milk relative to soy merits further research.

## Supporting information

S1 Data Database of the children included in the study. (DTA)

S1 Text Consolidated Standards of Reporting Trials (CONSORT) checklist. (PDF)

S2 Text Treatfood study protocol. (DOC)

## Abbreviations

CHW: community health worker
CSB: corn-soy blend
D_2_O: deuterium oxide
DS: dehulled soy
DSM: dry skimmed milk
FFM: fat-free mass
FFMI: fat-free mass index
FM: fat mass
FMI: fat mass index
ITT: intention-to-treat
LNS: lipid-based nutrient supplement
MAM: moderate acute malnutrition
MUAC: mid-upper arm circumference
PP: per protocol
RUTF: ready-to-use therapeutic food
SAM: severe acute malnutrition
SI: soy isolate
TBW: total body water
WHO: World Health Organization
WHZ: weight-for-height *z*-score
