# Supplementary material for: Effectiveness of food supplements in increasing fat-free tissue accretion in children with moderate acute malnutrition: A randomised 2 × 2 × 3 factorial trial in Burkina Faso
Source: PLoS Med. 2017 Sep 11;14(9):e1002387. doi: 10.1371/journal.pmed.1002387 (PMC5593178; doi:10.1371/journal.pmed.1002387)
Supplement: S1 Text — (PDF) [file pmed.1002387.s002.pdf]

This supplement contains the following items:

1. Original final protocol, incl. statistical plan
2. Summary of changes to the protocol
3. Summary of changes to the statistical plan

Effectiveness of improved diets for children with moderate acute malnutrition:
a randomized controlled trial in
Province du Passoré, Burkina Faso

As part of the

TreatFOOD research project

(Treatment of childhood undernutrition: development of and access to improved foods)

Title:	Effectiveness of improved diets for children with moderate acute malnutrition: a randomized controlled trial in Province du Passoré, Burkina Faso
Version:	52st Draft, English version, August 2013[1]
Study Site:	Province du Passoré, Burkina Faso.
Funding	Médecins Sans Frontières<br>DANIDA, the Danish International Development Assistance
Study Type:	Randomized control trial
Sponsor:	Department of Nutrition, Sports and Exercise, SCIENCE University of Copenhagen, Denmark[2]
Principal Investigator:	Prof. Henrik Friis, Department of Nutrition, Sports and Exercise, SCIENCE University of Copenhagen, Denmark[3]
Co-Principal Investigator(s):	Prof. Kim Fleischer Michaelsen, Department of Nutrition, Sports and Exercise, SCIENCE University of Copenhagen, Denmark<br>Dr Vibeke Brix Christensen, Médecins Sans Frontières-Denmark & Dept of Paediatrics, Rigshospitalet, Denmark<br>Professor Nikki Blackwell (University of Queensland), Medical Director Alima<br>Dr Isabelle Defourny, Director of Operations, Alima
Co-Investigators:	Bernadette Cichon, Médecins Sans Frontières-Denmark<br>Dr Christian Fabiansen, University of Copenhagen, Denmark<br>Ann-Sophie Iuel-Brockdorff, Médecins sans Frontières-Denmark<br>Dr Maren Rytter, University of Copenhagen, Denmark,<br>Associate Professor Christian Ritz, University of Copenhagen, Denmark<br>Dr Adama Konate, ALIMA, Burkina Faso<br>Charles Yameogo, ALIMA, Burkina Faso
Partners:	Médecins sans Frontières Denmark<br>Department of Human Nutrition, University of Copenhagen<br>ALIMA, The Alliance for International Medical Action, Dakar, Senegal<br>Institut de Recherche en Sciences de la Santé, Burkina Faso

Technical referents for protocol development:

Dr André Briend, Tampere University, Finland

Dr Martin Bloem, Policy, Strategy and Program Support Division, World Food Program

Dr Saskia de Pee, Senior Technical Advisor Nutrition and HIV/AIDS, World Food Programme

Prof. Suzanne Filteau, London School of Hygiene & Tropical Medicine, UK

Prof. Per Ashorn, Tampere University, Finland

John Wood, World Food Program, Rome, Italy

Dr. Jonathan Wells, Institute of Child Health, UK

---

[1] The Parties have decided to change the sponsor of the Treatfood Project meaning that that it is the University of Copenhagen that shall take over the responsibility of sponsor for the Treatfood Project and that MSF Denmark shall no longer be the sponsor of the Treatfood Project.The Ethical Committee in Burkina Faso has been informed about this change. The latest version of the protocol contains the relevant changes related to the change of sponsorship. .

[2] See CV in annex 1

[3] See CV in annex 1

Anne-Louise Hother Nielsen, University of Copenhagen, Denmark
Dr. Daniel Faurholt-Jepsen, University of Copenhagen, Denmark
Line Brinch Christensen, University of Copenhagen, Denmark

List of abbreviations

AGP	Alpha-acid-glycoprotein
APP	Acute phase protein
BIA	Bioelectrical impedance analysis
CIOMS	The Council for International Organizations of Medical Sciences
CRP	C-reactive protein
CSB	Corn Soy Blend
CSPS	Centre de Santé et de Promotion Sociale
CRF	Case-report form
DSM	Dry Skimmed Milk
EFA	Essential fatty acids
FAO	The Food and Agriculture Organization of the United Nations
FBF	Fortified Blended Flours
FGD	Focus Group Discussion
IAEA	International Atomic Energy Agency
ICH-GCP	International Conference on Harmonisation- Good Clinical Practice
iCSBS	Improved Corn Soy Blend Supplement
IGF-1	Insulin-like Growth Factor 1
IRSS	Institut de Recherche en Sciences de la Santé, Burkina Faso
LNS	Lipid-based Nutritional Supplement
MAM	Moderate Acute Malnutrition
MSF	Médecins sans Frontières
MUAC	Mid Upper Arm Circumference
RCT	Randomized Controlled Trial
RUTF	Ready-To-Use-Therapeutic Food
Science	The Faculty of Science, University of Copenhagen
SAM	Severe Acute Malnutrition
TBW	Total Body Water
UNHCR	United Nations High Commissioner for Refugees
UNICEF	United Nations Children's Fund
WFP	World Food Program
WHO	World Health Organization
WHZ	Weight-for-Height Z-score

Summary

Background

Moderate acute child malnutrition (MAM) is a major global health problem, affecting about 36 million children worldwide. There is no consensus on how to manage children suffering from MAM. Children have typically been given fortified blended flours, such as corn-soy blend (CSB), a product developed more than 30 years ago. More recently the limitations of CSB have been recognized and WFP has developed and is currently introducing improved CSBs called CSB+ and CSB++. Other organizations are also currently developing and testing various products for the management of MAM, including lipid based nutrient supplements. However there is no consensus on the most suitable product. Therefore there is a crucial need to develop and evaluate specific interventions for children suffering from MAM.

New food products should be tested using outcome measures of importance for body function, rather than just increase in weight. Increase in weight may simply be due to accumulation of fat and therefore not providing a long-term benefit to the child. Randomized trials with lean body mass (LBM) as the primary outcome[1] and functional measures as secondary outcomes are needed to identify new cost-effective food supplements for MAM management.

Objective

Twelve investigational food supplements will be developed, including six improved corn-soy-blend based supplements (iCSBS) and six Lipid based Nutrient Supplements (LNS) combining different soy qualities (dehulled soy, soy isolate) and different amounts of total protein (0%, 20% or 50%) provided by dry skimmed milk (DSM)[2].

This trial aims to assess the effectiveness of a 3 month supplementation with these newly developed products for the management of children 6-23 months old with MAM in Burkina Faso. Effectiveness will be assessed by determining their effects on accrual of lean body mass (primary outcome), linear growth, physical activity, motor milestones, morbidity, hemoglobin concentration, serum acute phase proteins, IGF-1, serum ferritin, serum

[1] The deuterium dilution technique is recommended as the technique of choice to measure the lean body mass in infants and young children *(Wells et al, 2006 & 2009, IAEA)*. The technique consists in giving water labeled with deuterium (a stable, naturally-occurring isotope of hydrogen) and to measure, after equilibration, the contents of deuterium in a saliva sample. This measurement allows estimating the total body water (TBW). With the estimation of the TBW, it is possible to estimate the amount of LBM from the hydration of the fat free mass (*IAEA)*).

[2] 0%, 20% and 50% of total protein provided by DSM corresponds to 0%,8% and 20% of total weight.

transferrin receptor, retinol binding protein, whole blood concentrations of essential fatty acids (secondary outcomes) and thymus size. Their respective acceptability will also be assessed.

Methods

Trial setting and period

This trial will be carried out in five Centres de Santé et de Promotion Sociale (CSPS) supported by ALIMA in the province of Passoré in the northern part of Burkina Faso. The trial is expected to begin in August 2013. The trial has been preceded by a pilot study in February 2013 aiming to test all tools and procedures, to evaluate the acceptability of the experimental foods and to explore perceptions, knowledge and beliefs of caretakers about malnutrition. Formative research about diets and feeding practices and food availability has also been carried out during the pilot in order to adapt locally available nutrition education materials..

Participants

All children arriving spontaneously at the CSPS program sites will be screened for eligibility. Furthermore active screening will be done in the villages by community health workers supervised by ALIMA. Children will be eligible for inclusion if they fulfil the following criteria:

- 6-23 months of age[1]
- weight-for-height z-score (WHZ) ≥ -3 and < -2 (WHO growth standard) or
- MUAC ≥115mm and < 125 mm,
- resident in the catchment area at the time of inclusion,
- whose parents/guardians consented to participate.

Children suffering from or having received treatment for severe acute malnutrition (WHZ < -3, mid-upper-arm circumference <115 mm, oedema) in the last two months, children with medical complications requiring hospitalization or having been hospitalized in the last two months and children with known allergy to milk, peanut, CSB and/or RUTF will be excluded.

Trial design

The study consists of a randomized controlled trial of 12 parallel groups each. In total, 1608 eligible children will be included, 134 in each of the 12 intervention groups.

[1] A local calendar of events will be piloted. If it does not appear suitable for inclusion based on age, children would be included based on a combination of event calendar and height.

Figure 1. Trial Design

		Dry skimmed milk (% mass / %protein)			
		**0%**	**8/20%**	**20/50%**	
R	**CSB/Dehulled soy**	a	b	c	1.
	**CSB/Soy isolate**	d	e	f	
	**LNS/Dehulled soy**	g	h	i	2.
	**LNS/Soy isolate**	j	k	l	

Interventions

The intervention will last for three months, with an additional three months of follow up. Children will be individually randomized to either: 1) newly developed iCSBS (500 kcal daily for 3 months); or 2) newly developed LNS (500 kcal daily for 3 months). In each of the iCSBS and LNS arms, children will be further randomized to a supplement based on dehulled soy or soy isolate and containing either 0%, 20% or 50% of total protein provided by DSM.

In addition, all groups will receive health and nutritional education and a fortnightly medical evaluation for the three months of intervention and monthly medical evaluation during the three months of follow-up. Children that have not recovered from MAM[1] after 3 months of intervention or who are losing more than 5% of their weight from admission on two consecutive visits will be referred for medical evaluation at Yako Hospital.

Outcomes

Primary outcome: absolute lean mass increment from baseline to 3 months (deuterium dilution method). ***Secondary outcomes:*** increase of WHZ to >-2, linear growth, increase in MUAC > 125 mm, physical activity, motor and language milestones development, morbidity, level of hemoglobin, serum acute phase proteins, insulin-like growth factor-1, serum ferritin, thymus size and essential fatty acids, acceptability of and adherence to the products.

[1] That is, children who did not reach a WHZ ≥ -2 or a 15% weight increase if admitted with a MUAC ≥115mm et < 125 mm

Follow-up chart of the participants

Time (months)	0		1		2		3		4		5		6
Fortnightly visits	X	X	X	X	X	X	X	X	X	X	X	X	X
Screening for eligibility, recruitment, consent, randomization, baseline questionnaire (socio-demographic and medical characteristics)	○												
Food supplementation (LNS & iCSBS arms)	←---	------	------	------	------	------	---→						
Education on nutrition, hygiene and health	○		○		○		○						
Regular visits (clinical examination; morbidity, adverse events, and adherence questionnaire)	○	○	○	○	○	○	○		○		○		○
Weight, MUAC, skinfolds, knee-heel length	○	○	○	○	○	○	○		○		○		○
Height, crown-rump length	○		○		○		○		○		○		○
Blood sampling	●						●						●
Lean Body Mass (deuterium dilution test)	○						○						
Physical activity	○						○						○
Child development (motor and language milestones)	○						○						○
Acceptability questionnaire		○					○						
Diet questionnaire	○						○						○
Thymic size (Ultrasound)	○						○						○

Content

1. Introduction 10
   1.1. Background 10
   1.2. Investigational food supplements 13
   1.3. Outcomes measures 15
   1.4. The context of Burkina Faso 17
   1.5. Rationale 19
2. Objectives 20
   2.1. General objective 20
   2.2. Specific research objectives 20
3. Methods 21
   3.1. Effectiveness randomized controlled trial 21
      3.1.1. Study setting and study period 21
      3.1.2. Study population 21
      3.1.3. Interventions: food products and quantities, medical treatment and incentives 22
      3.1.4. Study design 24
      3.1.5. Outcomes and adverse events 25
      3.1.6. Study withdrawals and referrals 26
      3.1.7. Sample size 27
      3.1.8. Randomization, sequence generation, allocation and concealment 27
      3.1.9. Blinding 28
      3.1.10. Participants follow-up chart 28
      3.1.11. Data collection 30
      3.1.12. Data management 35
      3.1.13. Data analysis 36
      3.1.14. Strengths and Limitations to the study 37
   3.2. Child care practices related to supplementary feeding and the perception, acceptability of and adherence to the food 38
4. Ethical and administrative considerations 40
   4.1. Authorizations 40
   4.2. Informed Consent 40
   4.3. Risks and benefits 41
   4.4. Food producers 42
   4.5. Commitment of ALIMA to use the results of the trial and sustainability of the intervention .. 43
   4.6. Collaborative partnership 43
   4.7. Financing and Insurance 43
   4.8 Communications 43
5. Study schedule and training 44
6. References 45

1. Introduction

1.1. Background

Acute child malnutrition (wasting) is a major global health problem, resulting in considerable morbidity and mortality. Children surviving from malnutrition may grow up with impaired intellectual and working capacity (*Black RE et al, 2008*). It has been estimated that more than 19 million children worldwide suffer from severe acute malnutrition (SAM, defined by a weight-for-height < -3 z-scores), and, in addition, more than 36 million (*Black RE et al, 2008*)[1] from moderate acute malnutrition (MAM, defined by a weight-for-height between -3 and -2 z-scores), which may progress to SAM.

Research has advanced the treatment of SAM, and significantly reduced its case fatality due to improved management protocols (*Manary et al, 2008*) using special milk based formulas (F-75 and F-100) and lipid-based Ready to Use Therapeutic Foods (RUTFs). In contrast to SAM, the management of MAM has only recently started to get more attention. Children with MAM have typically been given fortified blended flours (FBF) made by mixing soy and maize flours with added vitamins and minerals, such as corn-soy blend (CSB). The impact of this approach has been regularly questioned over the last 30 years (*Beaton GH et al, 1982; Navarro-Colorado C, 2007*). Until recently, their apparent lack of impact has been attributed to programmatic factors, such as food sharing, or the effect of repeated infections limiting the impact of food supplementation. These limitations have been challenged by the success of treating and preventing MAM with RUTF in conditions where the same programmatic difficulties applied *(Defourny I et al, 2007; Isanaka S et al, 2009; Nackers F et al, 2010)*.

FBF were designed more than 30 years ago, at a time where an insufficient protein intake was perceived as *the* major problem in child nutrition in low-income countries *(Prentice AM, 2005; McLaren DS et al, 1982)*. As a result, they have a higher proportion of energy derived from proteins than foods currently used to treat SAM (F100 and RUTF), which may be unnecessary and may decrease appetite (*Halton TL et al, 2004*). This high level of protein is often obtained by the inclusion of unrefined soy flour, which contains high levels of antinutrients, including phytates, that inhibit the absorption of key minerals needed for recovery, in particular zinc (*Erdman et al, 2004*). In addition to containing too much protein, FBFs have also been criticized because they do not contain animal-source protein *(Webb 2011).* Moreover, a large proportion of the phosphorus provided by soy flour is also provided by phytates and, as such, is not biologically available *(Golden M, 2008)*. In addition, soy

[1] Numbers are based on analysis of 388 national surveys from 139 countries carried out in 2005 and according to the new WHO growth standards (Black et al 2008).

flours may result in high levels of unabsorbed carbohydrates, which may provoke flatulence and decrease the acceptability of soy containing products *(Suarez et al, 2009)*. Another problem is that they do not meet nutritional needs of young children *(Chaparro and Dewey 2010).* Limitations of the formulation of the traditional CSB for the management of MAM have increasingly been recognized in recent years (*de Pee et al, 2009; WFP 2008).* Several organizations have started developing and testing new products for the treatment of MAM, either in the form of improved FBFs, such as CSB+ and CSB++ produced by WFP or lipid based products (based on RUTFs used for SAM treatment) called lipid based nutrient supplements (LNS) or Ready to Use Supplementary Foods (RUSF). For the purpose of this protocol, LNS will be used.

The interest in LNS for the management of MAM is a result of demonstrated success of RUTF for MAM management *(Defourny I et al, 2007)* and prevention *(Isanaka S et al, 2009).* While RUTFs appeared to be a good product for treating MAM it is also an expensive product and its price prevents the use beyond small-scale projects. Formulation of similar products, based on less expensive ingredients and given in smaller amounts, grouped under the generic term of lipid-based nutritional supplements (LNS) therefore seemed to be an attractive option. Promising results in terms of their acceptability (*Adu-Afarwuah S et al, 2010; Hess S et al, 2010)*, effect on growth (*Adu-Afarwuah S et al, 2007; Phuka et al 2008; Phuka et al 2009)* and weight gain (*Thakwalakwa c et al, 2010; Matilsky et al, 2009*) have already been demonstrated. They have also been shown to be more effective than CSB (*Matilsky et al, 2009*) and micronutrient supplements (sprinkles and nutritabs) (Adu Afarwuah et al, 2007). The higher quality CSBs may however be an alternative to LNS. One recent study has shown that CSB++ was not inferior to LNS in terms of recovery from MAM (LaGrone et al, 2012).

Some key factors contributing to the effectiveness of RUTF also characterize LNS, in particular:

- Their convenience of use: LNS can be eaten as a snack, without preparation, or added to family foods. LNS should not be mixed with water and are delivered in individual sealed packages preventing bacterial contamination.
- Their high fat content, including high levels of essential fatty acids: this may explain their efficacy, as low essential fatty acid intakes, especially of the n-3 family, have been reported to limit growth of children in some African countries *(Rocquelin G et al, 2003)*.
- Their content of dairy products: milk apparently contains growth factors, which may be interesting, particularly in children with associated stunting (low height for age) (*Hoppe C*

et al, 2008); also, dairy products provide highly available phosphorus, and they are free of anti-nutrients.

There is a need to disentangle which factor is really important in RUTF to promote rapid catch-up growth, as each of these factors has different cost implications if formulated within a LNS or CSB. In particular, milk appears to be important during nutritional rehabilitation and is now added to an improved CSB, CSB++ (currently known as Supercereal+), designed for the use in small children. The addition of milk to CSB++ may also be responsible for the early successes of CSB++ (see above). Milk is however an expensive ingredient and the amount needed is unknown (*Michaelsen KF et al, 2009*). For example, adding 10- 15% milk powder to a FBF would double its price *(Hoppe C et al, 2008).* It is therefore important to identify whether milk is really necessary in both CSB and LNS products and if yes, how much milk is needed to achieve the best balance between effect (benefits in terms of functional outcomes in children with MAM) and price, *i.e.* the milk content that optimizes the cost-effectiveness of the product.

It is also possible to lower the anti-nutrient content of FBF by using soy protein extracts, instead of unrefined soy flours, to improve their impact. Soy protein extracts are used successfully in rich countries to prepare soy based infant formula with an efficacy to promote growth comparable to that of milk based infant formula (*Agostoni C et al, 2006*). Soy protein extracts could be a less expensive alternative solution as an ingredient in LNS or improved FBF. However, there is uncertainty about the efficacy of using plant protein isolates as a less expensive substitute for dairy products.

Existing formulations of LNS products for the treatment of MAM are varying in terms of composition. Some products are containing milk (Eezee RUSF from GC Rieber Compact A/S) while in others, milk is replaced by whey (PlumpySup from Nutriset) and only in negligible amounts. Furthermore the quality of soy is also varying in the different existing LNS used for the treatment of MAM today. Indeed, adding more milk than necessary will increase the cost of the product, and prevent some children from getting access to treatment.

It is therefore important to explore the different options in terms of quantity of milk and quality of soy needed for the treatment of MAM, both for LNS and FBFs, and compare their respective effectiveness and cost implications.

Although their nutritional value is the first consideration, the newly developed food supplements should also be consistent with cultural contexts. Providing food aid that is culturally appropriate to recipients and that can be used efficiently at household level is of

primary importance (*The sphere project*). The effectiveness of the supplementary products will depend, among other things, on its acceptability, adherence and the way the products are perceived and managed within the household, and these factors are therefore critical for future project implementation (*Santon IS et al, 2005; Flax VL et al, 2009; Adu-Afarwah S et al, 2008; Rowe JP 2008).* Furthermore, child survival, growth and development depend not only on food intake, but on multiple direct and indirect factors as described in UNICEF's conceptual framework of malnutrition *(UNICEF 1990)*. As an example, the amount of food a child consumes is not only determined by the amount and quality offered, but also on the behavior of the caregiver *(Bentley MB et al, 1991)*, and child care practices related to feeding are important determinants for dietary intake and ultimately child growth. A well planned approach based on an understanding of the context is crucial prior to program planning and implementation, and may lead to better and more effective provision of nutrient supplementation programs. Therefore, this study will also focus on childcare practices related to supplementary feeding as well as perceptions, acceptability of and adherence to new and improved diets.

1.2. Investigational food supplements

Twelve investigational food supplements will be developed based on current available evidence (*Michaelsen KF et al, 2009; Golden M, 2008)* and international guidance (WFP, WHO, MSF, ALIMA). These include improved CSB based supplements (iCSBS) and LNS combining different soy qualities and different amounts of total protein provided by dry skimmed milk (DSM)[1], as follows:

- 3 iCSBS based on dehulled soy, with either 0%, 20% or 50% of total proteins provided by DSM
- 3 iCSBS based on soy isolate, with either 0%, 20% or 50% of total proteins provided by DSM
- 3 LNS based on dehulled soy, with either 0%, 20% or 50% of total proteins provided by DSM
- 3 LNS based on soy isolate, with either 0%, 20% or 50% of total proteins provided by DSM

Thus a total of 12 products, 6 in the form of LNS and 6 in the form of CSB.

[1] 0%, 20% and 50% of total protein provided by DSM corresponds to 0%, 8% and 20% of total weight.

Both types of product will comply with the WHO technical note “Supplementary foods for the management of moderate acute malnutrition in infants and children 6 – 59 months of age”(WHO 2012)

The products will not have the same nutrient density per 100g, but will provide the same total amount of protein and micronutrients per daily serve (130g of CSB or 92g of LNS).

The fatty acid profile will be similar in all twelve products and the same supplier for the main raw material, dehulled soya and soy isolate, will be used for both LNS and CSBs.

A vitamin and mineral premix will be designed for all products and will meet the quantities specified in the WHO technical note. Some adjustments will be necessary between products because e.g. dry skim milk in larger quantities will be a significant contributor of e.g. calcium, potassium and phosphorous, and because the concentration of certain vitamins decreases during the preparation of CSB. The aim is a uniform micronutrient composition across all products for final consumption.

For a detailed description of the composition of the products, please see annex 2.

LNS will be provided in individual packaging, ready for consumption with no need forpreparation or cooking. All LNS individual packs will have similar packaging, whatever the type of soy or the milk content, and will only differ by a number.

iCSBS fortnightly food rations will be delivered to the caretakers in bags of 1.7 kg which corresponds to a bi-weekly ration (130 g/child/day). All iCSB bags will have similar packaging, whatever the type of soy or the milk content, and will only differ by a number. iCSBS have to be consumed as a porridge or gruel. The flour should be mixed with an appropriate proportion of clean water followed by a cooking time at simmering point from five to maximum ten minutes.

For children with MAM, it is usually recommended to provide a food supplement daily ration of 75 kcal/kg that covers the daily micronutrients requirements (*Manary et al, 2008*). In addition to the usual diet, this quantity is sufficient to promote catch up growth. However, in order for possible intra-household food sharing to be taken into account, many organizations usually distribute a food supplement providing at least 1000 kcal/day, which appears relatively high for children with MAM *(de Pee S et al, 2009)*. Children with MAM (weight-for-height ≥ -3 and < -2 Z-scores) and measuring between 65 and 87 cm are expected to weigh between 5.5 and 10.4 kg (WHO growth standard). These children would therefore need a food supplement daily ration of 413 kcal (5.5 kg * 75 kcal/kg) to 780 kcal (10.4 kg * 75

kcal/kg). In this study, children will receive a fixed daily ration equivalent to 500 kcal/day. The same amount of energy will be provided for all food supplements.

1.3. Outcomes measures

Previous studies have often assessed the effect of various foods supplements for MAM on so-called "*nutritional recovery", i.e.* increments in the weight-for-height indicator to above a certain value (*Nackers F et al, 2010; Matilsky DK et al, 2009; Ciliberto MA et al, 2005; Patel MP et al, 2005*). However, weight gain alone is a suboptimal indicator, as it may merely reflect accumulation of fat and therefore not a long-term benefit for the child. When assessing child nutritional improvement, standard anthropometric measurements, i.e. weight, height, mid-upper-arm circumference (MUAC), and kneeheel length, as a measure of short-term linear growth (*Doherty et al, 2002; Michaelsen KF 1997)*, should be taken into consideration. Furthermore, additional indicators are needed to better assess the quality of the recovery.

An important indicator is body composition, namely what components comprises a person's body weight. In the most simple body composition model, body weight is divided in fat-free and fat-body mass (*International Atomic Energy Agency (IAEA)*). Fat free mass or Lean Body Mass (LBM) is known to be associated with survival in starvation and acute illness (*Sauerwein HP et al, 2010*) and to be accompanied by beneficial functional outcomes *(Wells J et al, 2009, Roubenoff R et a, 1991*). Several methods allow for the assessment of body composition, although there is no gold standard in vivo. Some of these techniques (such as dual-energy x-ray absorptiometry, or densitometry by plethysmography) require specialized equipment and do not appear suitable for conducting research in children living in developing countries. One easy-to-use technique is the measurement of the skin fold thickness (of the arm and/or the trunk) as an estimation of subcutaneous fat depots. This has been used for decades (*Wells J et al, 2008*) and tables of "triceps and sub scapular skinfold-for-age z-scores" are included in the new WHO Child Growth Standards (*WHO 2007*). The ratio between the triceps and the sub scapular skin folds allows describing the relation between central and peripheral fat depots. However, skin-fold thickness does not provide direct information on the LBM and it suffers from low intra- and inter-observer reliability *(Wells JC et al, 2006*).

It is therefore reasonable to use a second technique, LBM can also be assessed indirectly by measuring the total body water[1] of a child. The total body water can be measured using a

[1] Water is the largest component of the body and is found exclusively in the fat free mass. Total body water includes both intracellular fluid and extra cellular fluid.

tracer consisting of heavy water[1] (deuterium oxide, ^2H_2O or D_2O). This tracer is naturally occurring in body water in small amounts, and total body water is assessed by measuring the concentration of the tracer in saliva samples from a child taken before and after oral administration of the tracer. With the estimation of total body water it is possible to estimate the amount of LBM from the hydration of fat free mass (*IAEA)*. The tracer technique is regularly used in human studies in both developed and developing countries, including West African countries such as Senegal (Dioum et al, 2005; Diouf et al, 2009) and appears to be appropriate for use in infants and young children *(Wells JC et al, 2006, IAEA)*. The University of Copenhagen has recent experience using this technique in developing countries such as Tanzania, Ethiopia and Cambodia (*Praygod G, 2013* and other yet unpublished studies).

Beneficial functional outcomes linked to nutritional status also include immune function, physical activity and child development. The immunodeficiency associated with malnutrition is the primary reason for children with malnutrition to be more susceptible to infectious disease, and more likely to die than their well-nourished peers. The nature of this immunodeficiency is only partly understood, but it is well established, that the size of a child's thymus is a useful indicator of the "immunodeficiency of malnutrition" *(Savino and Dardenne, 2010*). Malnourished children have small thymuses, and studies from West Africa have found a small thymus is a strong and independent risk factor for mortality (*Garly et al, 2008; Aaby et al 2002*). The size of the thymus can be assessed by ultrasound (*Jeppesen 2003*).
The level of physical activity may be an indicator of wellbeing in children and changes may affect body composition (*Reichert et al 2009).* Thus, associations between physical activity, weight change and changes in body composition during nutritional rehabilitation are important to evaluate. This can be assessed using an accelerometer (*Cliff DP et al, 2009*). Further, malnutrition may also alter cognitive development *(Victoria 2008)* and delay the achievement of motor and cognitive milestones of young children. This can be measured with a child development test such as the Malawi Developmental Assessment Tool (MDAT) recently validated in Malawi (*Gladstone et al, 2010*)).

In addition to anthropometric, LBM and functional outcome measures, biological indicators may also help assess changes in nutritional status. Blood hemoglobin and serum ferritin levels are used to evaluate the effect of nutritional interventions on anemia and iron status respectively (*Lopriore C et al, 2004; Kuusipalo H et al, 2006; Andang'o PE et al, 2007*).
In addition, nutritional status plays an important role in the regulation of the serum insulin-like growth factor-1 (IGF-1) and its variations may serve to monitor improvement in children's

[1] The method is a state-of-the-art method adapted to field use and recommended by the International Atomic Energy Agency (IAEA) in Vienna, Austria.

nutritional status, particularly in relation to linear growth (*Kouanda et al, 2009*). Since, the acute or chronic inflammatory status might influence the levels of ferritin (*Beard et al*, 2006) and IGF-1 (*Kouanda et al, 2009*), it is recommended to measure, simultaneously, serum concentrations of acute phase proteins (APP) such as slow reacting α_1-acid-glycoprotein (AGP) and the C-reacting protein (CRP). These APP are also objective markers of infections and have been shown associated with maternal reports of morbidity among Ghanian (*Filteau SM et al, 1993*) and Zambian (*Filteau SM, unpublished observations*) children.
Finally, essential fatty acids (EFA) are crucial for child growth and development (*Uauy et al, 2003*) and malnourished children may be EFA-deficient *(Squali Houssaïni FZ et al, 2001; Decsi T et al, 1998; Briend et al, 2011)*. Blood EFA levels can be used to assess the impact of dietary interventions on fatty acid status (*Fratesi et al, 2009; Briend et al, 2011*) and might therefore help evaluating child nutritional rehabilitation in this trial.

1.4. The context of Burkina Faso

Burkina Faso has approximately 17 million inhabitants. Child mortality in the country is high, with an average of 176 deaths per 1000 live birth in children less than 5 years old. The high child mortality rate is among other things due to malnutrition (*Black RE et al, 2008*).
According to a recent SMART survey carried out in August-September 2011(*Ministère de la Santé and Direction de la Nutrition, Burkina Faso, 2011*), the prevalence of Global Acute Malnutrition in Burkina Faso is 10,2% (95% CI 9,6-10,7%), which is high if compared to the 10% emergency threshold as defined by WHO.
Similarly to other countries in the Sahel region, Burkina Faso is affected by recurrent food insecurity and nutrition crisis . This was last seen in 2012 where late rains, reduced harvest and increased food prices (by 30-60% between January 2011 and January 2012) and left the population more vulnerable and prone to malnutrition.

The treatFOOD project will take place in the Province du Passoré also known as the health district of Yako (after the main city). It is part of the northern region together with the districts of Gourcy, Séguénéga, Titao et Ouahigouya and consists of 9 departments: Arbollé, Bagaré, Bokin, Gomponsom, Kirsi, Latoden, Pilimpikou, Samba et Yako.

The province covers an area of 4078 Km² and has 372 403 inhabitants including 70 017 children under 5 (Ministère de la Santé, District Sanitaire de Yako, 2011). The main city of Yako is about 110 Km from the country's capital Ouagadougou.

The district of Yako has 53 health centres and 1 small hospital. Each village has two agents de santé communautaires (community health workers) who support the health staff with some aspects of their work such as community sensibilisation and mobilization as well as health education.

In 2011 the malnutrition rates in the province were 11.8% (GAM) and 1.6% (SAM) and one of the areas identified to be at risk of food insecurity and a priority region for WFP.

From 2007-2011 the management of malnutrition in the province was ensured by Médecins sans Frontiers-France (MSF-F), who handed over their activities to the Ministry of Health in July 2011. Since July 2012, the NGO ALIMA has in collaboration with a local NGO KEOOGO been implementing a nutrition program in the same area in the province where MSF-F was previously working. The objective of the program is to support government health structures in the treatment of SAM.

ALIMA is working together with KEEOGO in supporting the 53 health structures in the district for diagnosis and outpatient treatment of SAM. ALIMA is furthermore supporting the in-

patient treatment of SAM at the paediatric unit at Yako hospital by reinforcing capacity and providing technical support. For more information on ALIMA and KEOOGO, please see annexes 3 and 4.

Recruitment and follow-up of children enrolled in the research project is planned to take place in five CSPS supported by ALIMA. In many of the CSPS, un-used structures from when MSF-F used to support activities, are present and could be used for the project, if adjusted and renovated slightly. Behind the research project is an experienced research team who will take turns being based in Yako for supervision and support. Furthermore local staff will be recruited and trained for the implementation of the research project, in addition to existing health staff at the CSPS.

1.5. Rationale

Nutrition is one of the main socio-economic and political issues of Burkina Faso and is a priority for the government in order for the Millenium Development Goals to be reached. The treatment of SAM has improved significantly in recent year while guidelines for the treatment of MAM have remained unchanged. In many countries including Burkina Faso, although guidelines here are in the process of being changed, treatment with CSB is still recommended for the management of MAM. However, CSB has been proven to be inadequate for the treatment of malnutrition in children due to among other things high levels of anti-nutrients, inadequate micronutrient and protein content as well as a high viscosity (*de Pee et al, 2009*).

Treatment of MAM is essential in a public health perspective and in some of the most affected regions in Burkina Faso, aid organizations are currently introducing improved CSBs (CSB+ and CSB++) and other products for the management of MAM, including lipid based nutrient supplements. However, there is no consensus on how to manage children suffering from MAM and which the best product is**.** New food products should be tested using outcome measures of importance for body function, rather than just increase in weight. Randomized trials with lean body mass as the primary outcome and functional measures as secondary outcomes are needed to identify new cost-effective food supplements for MAM management. In this respect, the present proposal aims at comparing the effectiveness of improved diets in the nutritional improvement of children with MAM in Burkina Faso, considering the cost of the experimental food supplements.

2. Objectives

2.1. General objective

To assess the effectiveness of newly developed supplementary iCSBs and LNS for the management of children 6-23 months old with moderate acute malnutrition in Burkina Faso, by determining their effects on accrual of lean body mass, linear growth, functional and biological outcomes.

2.2. Specific research objectives

Primary objective:

- To assess the effectiveness of a 3 month supplementation with newly developed iCSBs and LNS on the increase of lean body mass among children 6-23 months old with MAM in Burkina Faso.

Secondary objectives:

- To assess the effectiveness of a 3 month supplementation with newly developed iCSBs and LNS on weight-for-height increase, increase in MUAC, linear growth, thymic size, physical activity, child motor and language development, serum APPs, serum IGF-1, ferritin and hemoglobin, whole EFA blood concentrations and reported morbidity among children 6-23 months old with MAM in Burkina Faso.
- To compare the effectiveness of different soy qualities (dehulled soy versus soy isolate) and different amounts of protein provided by dry skimmed milk (DSM) on the outcomes among 6-23 months old children with MAM in Burkina Faso[1].
- To explore childcare practices related to supplementary feeding as well as the adherence to and acceptability of the food supplements.

Other secondary objectives:

- To suggest the most effective food and duration of supplementation for MAM management, according to age and nutritional status,
- To assess the level of baseline body composition, biological profile, physical activity and motor and language development of children 6-23 months old with MAM in Burkina Faso,

[1] By merging, in the LNS and the iCSBS arms separately, the dehulled soy groups and soy isolate groups on the one hand and the 0% DSM, 20% DSM and 50% DSM groups on the other hand.

- To identify anthropometric and other simple predictors of lean body mass,
- To compare the costs of the food products

3. Methods

3.1. Effectiveness randomized controlled trial

3.1.1. Study setting and study period

The trial will be conducted in selected district health centers “Centre de Santé et de Promotion Sociale” (CSPS) supported by ALIMA in the province of Passoré in the northern region of Burkina Faso. ALIMA’s program consists of support to district health structures for the management of severe acute malnutrition (SAM) in the District hospital of Yako and 53 CSPS in the province of Passoré. The trial will take place in five selected CSPS. Admission and follow-up take place 5 days a week on each site[1]. The recruitment of children for the trial is expected to begin in August 2013. The intervention is planned for three months, with an additional three months of follow-up.

The prevalence of HIV among adults in Burkina Faso is 1.2 %[2]. The estimated number of HIV infected children among our study participants is therefore likely to be very low.

3.1.2. Study population

Children will be eligible for inclusion if they fulfill the following criteria:
- weight-for-height z-score (WHZ) ≥ -3 and < -2 (based on the WHO growth standard)[3] or
- MUAC ≥115mm and < 125 mm
- 6 to 23 months of age[4],
- resident in the catchment area at the time of inclusion,
- whose parents/guardians have signed (or thumb-printed whenever illiterate) the informed consent.

[1] From 7.30am to 4 pm, all year long.
[2] UNAIDS 2009
[3] According to the workload, prior MUAC screening might be implemented.
[4] A local calendar of events will be piloted. If it does not appear suitable for inclusion based on age, children would be included based on a combination of event calendar and height.

Will be excluded:

- Children with severe acute malnutrition defined as a WHZ < -3, and/or a MUAC <115 mm, and/or bilateral pitting oedema. They will be referred to the health centres or Yako hospital for appropriate treatment,
- Children with medical complications requiring hospitalization,
- Children whose household plans to leave the catchment area in the next 6 months.
- Children with a hemoglobin concentration <4g/dl or with evidence of a decompensate anemia (eg, dyspnea, tachycardia...). These children will be transferred to the ITFC supported by ALIMA for appropriate treatment.
- Children who have been treated for SAM or who have been hospitalized in the last 2 months,
- Children with known allergy to milk, peanut, CSB and/or RUTF,
- Children with a severe disability limiting the possibility of investigations,
- Children enrolled in any other nutritional program or part of any other study conducted in the area.

Children with MAM who do not wish to participate in the study or who are excluded due to some of the above mentioned criteria, will be referred to the national supplementary nutritional program currently supported by WFP.

Appropriate free care, including referral to nutritional rehabilitation or pediatric care at Yako hospital (supported by ALIMA) when needed, will be offered to children who are non-eligible due to SAM or decompensated anemia.

Children will be recruited among children presenting spontaneously at the CSPS. All children taken to CSPS will be screened for eligibility. Furthermore active case finding within the community by community health workers supervised by ALIMA will be done.

3.1.3. Interventions: food products and quantities, medical treatment and incentives

Food products and quantities

Children will receive 500 kcal/day. Food will be distributed fortnightly during a 3 month period. At each visit, a ration will be given on the spot. Mothers will be asked to bring back the empty LNS packs at the next visit to give to the personnel charge of the supplement (not to staff involved in measurements, to ensure blinding).

CSB will be provided in bi-weekly rations, to be prepared at home.

During the entire follow-up, any child developing severe acute malnutrition (WHZ less than – 3 z-scores, MUAC <115 mm or edema) would be referred for treatment of SAM. The following children will be referred to Yako pediatric unit, which is supported by ALIMA, for medical evaluation;

- Children admitted based on WHZ ≥ -3 - and < -2 z-scores who have not reached a WHZ≥ -2 z-scores at the end of the 3 months intervention
- Children admitted on MUAC ≥115mm and < 125 mm who have not reached 15% weight gain,
- Children presenting a weight loss ≥ 5% from his/her weight at baseline for two consecutive visits.

Should these children receive other nutritional intervention during their hospitalization, they will be withdrawn from the study as failures to respond to treatment. Data obtained after receiving other nutritional intervention than previewed will not be included for data analysis.

Health and nutritional education

Education on nutrition, health and hygiene will be provided in group sessions to all caretakers of eligible children on a regular basis. The content of nutrition education sessions will be based on locally available material, but adapted according to formative research carried out during the pilot study conducted in February 2013 where l information of food availability and food consumption was collected.

In addition, caretakers of children allocated to the LNS and the iCSBS groups will receive specific recommendations according to the food to be taken by the child (frequency, timing, and, for the iCSBs groups, preparation and cooking).

Medical treatments

At inclusion, all children will benefit from:

- Measles vaccination, if there is no evidence of previous vaccination[1]
- Treatment against intestinal parasites (200 mg of Albendazole for children of 4 to 8 kg; 400 mg for children of more than 8 kg).
- Diagnosis of malaria with systematic treatment based on Artesunate-Amodiaquine (following National Guidelines) in case of positive results to the rapid test.

[1] Children vaccinated at 6 months would receive a second injection at 9 months.

- Vitamin A supplementation (100 000 UI for children of 4 to 8 kg; 200 000 UI for children of more than 8 kg), if there is no evidence of vitamin A supplementation in the previous month.
  (See annex 5)

A nurse will examine the children at inclusion and at each fortnightly (3 months of intervention) or monthly follow-up visit (3 months of follow-up) for a total of 6 months and any disease diagnosed will be treated following National Guidelines[1]. Parents/guardians will be encouraged to bring their children to the clinic any time if the child is ill for medical assessment and appropriate treatment.

Incentives

All caretakers will receive a mosquito net at admission and a bar of soap every other week. At the end of the study (after 6 months), incentives will be given to all participants (likely clothing and a cooking pan). At the baseline, 3 months and 6 months follow-up visits (that is visits requiring the presence of the child during the majority of the day), children and caretakers will be provided with a meal and with activities during the waiting time (i.e knitting activities or others for the mothers and toys for the children). For evaluation of the level of physical activity, the children will be given a t-shirt to wear over the accelerometer, which can be kept after the study period.

3.1.4. Study design

Figure 3 *Trial design*

		Dry skimmed milk (% mass / %protein)			
		**0%**	**8/20%**	**20/50%**	
R	**CSB/Dehulled soy**	a	b	c	1.
	**CSB/Soy isolate**	d	e	f	
	**LNS/Dehulled soy**	g	h	i	2.
	**LNS/Soy isolate**	j	k	l	

[1] Children with moderate anemia (hemoglobin concentration <9gdl) would first receive treatment for possible associated infections. Hemoglobin concentration (Hemocue) would be assessed at the next visit and closely followed-up. Iron and folic acid supplementation would be considered if hemoglobin level does not improve with food supplementation and in the absence of associated infections.

Children will be randomized to either an iCSBs or a LNS intervention. Children randomized to the iCSBs arm will be further randomized to one of the 6 iCSBs: based either on dehulled soy or on soy isolate and containing either 0%, 20% or 50% of proteins provided by DSM. Similarly, children randomized to the LNS arm will be further randomized to one of the 6 LNS: based either on dehulled soy or on soy isolate and containing either 0%, 20% or 50% of total protein provided by DSM.

3.1.5. Outcomes and adverse events

Efficacy

- Primary outcome: absolute lean-mass increment (in kg) from baseline at 3 months using deuterium dilution technique.
- Secondary outcomes lean-mass increments in kg/cm and in g/kg/day, increase of WHZ to >-2, linear growth, MUAC, level of physical activity, development in motor and language milestones, thymic size, morbidity (e.g. malaria, severe anemia, severe acute malnutrition, diarrhea, respiratory infection, hospitalization, death), hemoglobin concentration, serum acute phase proteins, IGF-1, serum ferritin and whole blood concentrations of essential fatty acids.

Safety

Adverse events (AE)[1]: Although we do not expect the foods to cause any harm, peanut, milk, and soy proteins can be allergenic. AE could include: digestive symptoms (diarrhea and/or vomiting), cutaneous symptoms (hives) and, respiratory symptoms (wheezing) within one hour after ingestion of the food. The first dose of the products will be taken under observation and anaphylactic kits will be available at each recruitment sites, where the study nurse will be trained in how to respond to any allergic reaction. All signs of possible allergic reaction will be recorded as part of the fortnightly morbidity assessment, and will be treated accordingly. Serious AE related to this study could include: angioedema, anaphylactic reaction, and death. All serious AE will be immediately communicated to the trial manager who will ensure that they are addressed in an appropriate and timely manner and reported to all study investigators and trial sites, according to Good Clinical Practice. The trial manager will be responsible for the medical management of all adverse events, and will ensure referral to the national health system if necessary, including follow-up.

[1] Definition of the ICH-GCP glossary: Adverse Event : Any untoward medical occurrence in a patient or clinical investigation subject administered a pharmaceutical product and which does not necessarily have a causal relationship with this treatment. An adverse event (AE) can therefore be any unfavorable and unintended sign (including an abnormal laboratory finding), symptom, or disease temporally associated with the use of a medicinal (investigational) product, whether or not related to the medicinal (investigational) product. Serious Adverse Event (SAE): Any untoward medical occurrence that at any dose: results in death, is life-threatening, Requires inpatient hospitalization or prolongation of existing hospitalization, results in persistent or significant disability/incapacity, or is a congenital anomaly/birth defect.

Furthermore the national ethical review board will be notified and will be asked to advise the investigators of future actions to be taken.

In addition, any possible untoward medical event possibly related to the investigations conducted in this study (i.e. tracer dilution test, blood sampling) will also be collected during the investigation itself and as part of the morbidity assessment. Any research related conditions will be managed according to National Guidelines

3.1.6. *Study withdrawals and referrals*

Withdrawals will not be replaced. The reasons for withdrawal or switch to RUTF and their consequences on further intervention and follow-up are described in the Table 1.

Table 1. Reasons for withdrawal or referral.

Reasons for withdrawal [1]	***Further nutritional supplement***	***Further follow-up***	***Further assessment***
Withdrawals			
Spontaneous withdrawal of consent	None	None	None
Defaulters (having missed 2 consecutive visits), visited at home and not keen to come back in the study	None	Home visit : referral to the ATFC or health center in case the child developed SAM or any illness	- Life and nutritional status at the home visit.<br>- Reasons for defaulting.
Lost to follow-up (having missed 2 consecutive visits, visited at home but not possible to locate)	None	None after unsuccessful home visit	None
Serious Adverse Events	Local Foods	Until the end of treatment	- Outcome of the treatment<br>- Anthropometric measurements and clinical assessment until end of treatment
Other			
Transfer to the ATFC or ITFC,	RUTF or food as provided at the ITFC or the hospital	Until the end of treatment	- Outcome of the treatment<br>- Anthropometric measurements and clinical assessment until end of treatment
Hospitalization	Continue intervention	Until the end of treatment	- Medical follow-up Outcome of the treatment<br>- Anthropometric measurements and clinical assessment until end of treatment[2]

[1] Participants who withdraw informed consent or were lost to follow-up will not receive further incentives. Other participants will receive the incentives according to the planned timing at the start of the study.

[2] Children receiving other nutritional intervention (which is not part of Study) after being referred, they will be considered as failure to respond to treatment, and data obtained after receiving other nutritional intervention than previewed will not be included for data analysis

Appropriate free care, including referral when needed, will be ensured by the study team and ALIMA to children who develop SAM during the study. Children diagnosed with other diseases during the study will be referred for relevant treatment. Referral and treatment within the scope of care of the national health system in Yako will be provided free of care.

3.1.7. Sample size

In the absence of information on the increase of LBM from 0-3 months among children with MAM in this age group, we assume that a 0.4 SD difference in change/increase in lean body mass between groups as clinically relevant. The sample size per group, n, required to detect a change, d, in a continuous variable with standard deviation, s, with alpha set at 5% and a 80% power, is $16*s^2/d^2$. Hence, to detect a 0.4 SD greater increase in lean body mass between 2 products/cells, we will need 100 children in each group. To allow for a 25% expected loss to follow-up/drop-out in the nutritional program in Burkina Faso, we will include 134 children in each of the 12 intervention groups (cells) then 12x134=1608 children.

Considering a recruitment rate of 54 children with MAM per month per CSPS, the recruitment is therefore expected to last about 6 months and will be limited five CSPS (i.e., 54 children with MAM * 6 months * 5 CSPS = 1620 children).

3.1.8. Randomization, sequence generation, allocation and concealment

Each of the twelve food products (six CSB and six LNS) will be randomly letter coded by GC Rieber Compact by the letters M, N, P, Q, R, S ,T, U, V, X, Y, Z, ("O" is omitted due to similarity with "Q"). Two identical documents (code documents) stating which product corresponds to which code is prepared and placed in sealed envelopes also by GC Rieber Compact. The code documents are kept in safety boxes, one at the MSF-DK office, and one at the Dept. of Nutrition, Sports and Excercise, University of Copenhagen. The code will first be revealed following the primary analysis.

Stratified, blocked randomization will be used to randomly allocate children to the twelve experimental foods. Stratification will be done by recruitment site (CSPS), ie, separate random sequences will be made, one to be used at each of the five sites. This will ensure that site, which may be strongly associated with the outcome and thus an important potential confounder, will be equally distributed between the intervention groups. Blocked randomisation will be used, with varying block size, to ensure that for each site, children will

be allocated evenly to the trial arms. The random sequences (RS) are made using www.randomization.com.

Sealed envelopes will be labelled with the unique patient code and will contain the intervention allocated to the patient. The envelopes will be opened only after the completion of the screening and the final recruitment of the patient, ensuring concealment.

In case two or more siblings are presenting MAM, only one child per mother will be included in the trial (i.e. the first child diagnosed with MAM or, if several siblings are diagnosed on the same day, one of them will be selected by simple random sampling). The other siblings will however benefit from the same nutritional intervention as her/his brother/sister (whether it is, the iCSBs or LNS group). For these siblings, no specific assessments would be done except the routine medical evaluation and standard anthropometric measurements.

3.1.9. Blinding

This is an open trial since participants will not be blinded with respect to main type of food (LNS or iCSBs). However, within the arms with either iCSBs or LNS, participants will be blinded to the soy and milk content of the food. The investigator measuring the primary outcome (lean body-mass using tracer dilution) will be kept unaware of the allocation group and will therefore be blinded with respect to main type of food. In addition, the investigators measuring other biological factors, physical activity and milestones development will be blinded with respect to main type of food.
After baseline data have been collected, the child will be referred to the Supplement Responsible (SR). Only the SR will have access to the random sequence list.
For further information on the randomization and management of the experimental products, please see Standard Operating Procedure in annex 6

3.1.10. Participants follow-up chart

Active screening will be done within the community and children 6-23 months presenting at the CSPS will be assessed for eligibility. The caretakers of those eligible will receive detailed information by the study staff about the trial (see information notice in annex7) and will be invited to freely consent to participate. They will be asked to sign (or thumb-print if illiterate) the informed consent form (see in annex 8). An independent literate witness (from the community) will be required to be present during the reading of the consent form and sign in addition to the fingerprint requested from a non-literate study participant. After written informed consent has been obtained, children will be assigned a sequential study number.

This number will be reported on all the participant documents as well as on an identification bracelet and an identification card (that will include a picture of the child and his/her caretaker). Furthermore participation will be registered in the patient health card, to avoid that the patient seeks nutritional supplementation or treatment from other CSPS.

The participants will be assessed as described in the following Table 2. A participant will be considered as having missed the fortnightly or monthly visit if they present with a delay of more than one week. If the patient does not show up at the centre, after more than one week he/she will be visited at home to evaluate reasons for defaultering. Defaultering due to death will be reported with morbidity data

Table 2. Follow-up chart of the participants.

Time (months)	0		1		2		3		4		5		6
Fortnightly visits	X	X	X	X	X	X	X	X	X	XX	X	X	X
Screening for eligibility	○												
Recruitment/consent/randomization	○												
Baseline questionnaire (socio-demographic and medical characteristics)	○												
Food supplementation (LNS & iCSBs arms)	←--------------------------→												
Education on nutrition, hygiene and health	○		○		○		○						
Regular visits *(Questionnaire including morbidity and possible adverse events, clinical examination[1] by a nurse and adherence questionnaire)*	○	○	○	○	○	○	○		○		○		○
Anthropometry: weight, MUAC, knemometry and triceps- and subscapular skinfold	○	○	○	○	○	○	○		○		○		○
Anthropometry: height, crown-rump length	○		○		○		○		○		○		○
Blood sampling *(Hemoglobin, malaria, serum AGP, CRP, IGF-1, micronutrient status, essential fatty acids)*	●						●						●
Lean Body Mass (deuterium dilution test)	○						○						
Physical activity	○						○						○
Child development (milestones)	○						○						○
Acceptability questionnaire		○					○						
Dietary questionnaire		○					○						○
Thymic size (Ultrasound)	○						○						○

[1] Including malaria rapid test and hemoglobin measurment using the hemocue if necessary.

3.1.11. Data collection

In February 2013 the TreatFOOD pilot study was carried out in the Province de Passoré, to assess the feasibility, acceptability and best practice of a range of the methodologies scheduled to be used in the main study. The methodologies evaluated were:

1. Anthropometry (weight, height/length, MUAC, crown-rump length, knee-heel length, skinfold thickness)
2. Physical activity using Actigraph accelerometers
3. Deuterium dilution technique for the assessment of lean body mass, including saliva sampling
4. Child development using an adapted version of the Malawi Development Assessment Tool

The procedures of the different methodologies mentioned above and described below have been adapted according to results of the pilot and therefore the study context.

Reports from the pilot study are available upon request.

Anthropometric measurements

Weight, height, MUAC, crown-rump length, triceps skinfold, subscapular skinfold and knee-heel length will be measured for all children. All anthropometric measurements will be taken every second week during the intervention period except for height that will only be measured once a month. During the follow-up period, all measurements will be done monthly.

During the pilot study, the feasibility and acceptability of all measurements have been assessed. Furthermore the quality of training and measurements was assessed. The pilot study showed that all measurements were feasible in the study context and that procedures were well accepted by both mothers and children with few adjustments. Furthermore the quality of measurements of the pilot staff was acceptable after few days of training.

During the trial weight will be measured to the nearest 100 grams using an electronic scale that has a double weighing function. The double weighing function will enable young children to be on their mother's arm during weighing.. The scale will be regularly calibrated against a standard weight. Length or height and crown-rump length will be measured to the nearest 1mm using a standard UNICEF wooden measuring board. Children aged less than 24 months will be measured lying down and older children will be measured standing up. Knee-heel length will be measured to the nearest 0.1mm using an electronic hand-held

knemometer (*Michaelsen KM et al, 1991*). MUAC will be measured on the left arm to the nearest 1 mm.

Triceps and subscapular skinfold thickness will be measured using a Harpenden skinfold caliper to the nearest 0.2 mm. All measurements will be recorded twice, except for knee-heel length, which will be measured five times[1]. All measuring tools will be checked daily for accuracy and replaced if needed.

Physical activity assessment

Data on physical activity will be collected at baseline and at 3 and 6 months. The measurement will be done using a high frequency accelerometer (Actigraph). This is a harmless electronic device, which is dust and waterproof, and placed on the body with an elastic belt. It has previously been used extensively for studying physical activity in adults, adolescents and children (De Vries SI *et al*, 2006), but very limited data is available on its use in small children (Cliff DP *et al*, 2009), and to our knowledge no studies have been conducted on children recovering from MAM in West Africa.

Therefore, prior to the main study, a pilot study was conducted to assess how the device is perceived and tolerated in this context. The pilot showed that the accelerometer was well acceptedby children (20), mothers and the community and it was carried and returned after five days as instructed.

For the main study, the device will be placed on the waist of the child and should be carried at all times for 7 days at each assessment**,** to evaluate the level of everyday physical activity in the normal surroundings of the child. Children will be given a t-shirt to wear over the accelerometer. Upon completion of each measurement period the accelerometer will be taken off, and data transferred from the accelerometer into a computer for further analysis, using the actigraphy and management platform of Actigraph, ActiScience 5.

Tracer (Deuterium) dilution technique

Body composition will be assessed at baseline and after 3 using the tracer dilution technique. In this technique, children will be weighed and a saliva sample will be collected with a cotton swab. Then, all children will be given a fixed standardized oral dose of liquid tracer (Deuterium, D2O), using 5 gr D2O diluted in 10 ml ((~50% dilution) .. A second saliva sample will be taken after an equilibration time necessary for the tracer to evenly mix throughout the

body water. In healthy participants, equilibration is usually achieved after 2.5-4.5 hours and in general, children have faster water turnover than adults. However various disease states can influence water turnover and may delay equilibration (*IAEA*). Analysis of samples from the pilot study is ongoing and will determine the required equilibration time for children with MAM. During the equilibration time, children and caretakers will be provided with a meal and with activities (i.e knitting activities or others for the mothers and toys for the children). Health promotion sessions will also be conducted during this time.

The saliva sample will be analyzed for tracer distribution taking into account the volume of any fluid intake during the equilibration period. The concentration of the tracer in the saliva will allow estimating the total body water. Raw values for total body water will then be converted to lean mass measured in grams, using age-and sex-specific values for the hydration of lean tissue (*Fomon SJ et al,* 1982). The tracer enrichment of the saliva samples will be assessed using Fourier transform infrared (FTIR) spectrophotometry. Saliva samples will be analyzed at the IAEA Collaborating Centre, SJRI.

Child development

Child development will be assessed in all children at baseline and after 3 and 6 months using a piloted and adapted version of the Malawi Developmental Assessment Tool (MDAT) (*Gladstone et al., 2010*). The tool will focus on gross and fine motor development as well as language development and includes 30 milestones in each domain. Thus a total of 90 milestones

For the main study, testing of the child will take place in a quiet area. The child should be alert and well enough to be cooperative. If a child is too tired, irritable or sick to cooperate, the test will be postponed to the next fortnightly visit. The test will be carried out by trained health workers, in the presence of the mother/caretaker of the child. Health workers will evaluate each child starting with at least three milestones before the most age-appropriate milestone in each domain. . If the child passes these milestones, prior milestones will be rated as observed passed (10 points), Milestones will be rated with scores 10, 5 or 0. If the child can perform the milestone while being observed, the child will score 10. If the child cannot perform the milestone while being observed, but the mother reports that the child is able to perform the milestone at home, the child will score 5. If the child cannot perform the milestone and the mother reports that the child is not able to do so at home, the child will score 0.

After having failed six consecutive milestones in a domain (gross and fine motor or language), the assistant will move along to the next domain, following the same procedure.

After the evaluation, a total score will be calculated for each domain and an evaluation of the behaviour and cooperation of the child as well as the involvement of the mother will be carried out.

Questionnaire

At baseline, socio- demographic and medical characteristics of the participants will be collected using an interviewer-administered baseline questionnaire. At each subsequent fortnightly/monthly visit, caretakers will answer a questionnaire about morbidity. Some questions will also assess the adherence to the food supplement (quantities consumed). In addition, all data on morbidity as collected during the clinical examination by a nurse (symptoms, diagnosis and treatments prescribed) will be recorded in a case-report form. All serious AE will be immediately reported to the study supervisor. Childcare behaviors related to supplementary feeding and adherence to and acceptability of the products will furthermore be explored (as described in the section 3.3). The provisional list of information that will be collected is presented in annex 7. In addition dietary data will also be collected at inclusion, 3 and 6 months.

Blood sampling and analyses

Blood samples will be taken at baseline and thereafter at 3 and 6 months of follow-up.
An anesthetic crème will be applied to the skin prior to blood sampling to minimize pain.

2.5 ml of venous blood sample will be collected by standard phlebotomy. Directly after taking the blood sample 1 drop of whole blood will be put on filter paper with BHT – to be dried and stored in 4°C. Furthermore, 1 drop with be used to estimate hemoglobin concentration (HemoCue) on the spot and 1 drop will be used for malaria rapid-test on the spot (local standard).
The rest of the blood will be put into a tube and taken to the lab in Yako. At the lab the blood will be centrifuged and the serum added to small tubes, stored at -20ºC and then transported to the labs for analysis.

Since no laboratory in Burkina Faso performs all the desired analyses for research purposes, the samples will be exported for analysis.
- 0,2 ml of serum will be send to University of Copenhagen[1] for analysis of IGF-1.
- 0,2 ml of serum will be sent to Germany[1] for analysis of CRP, AGP, ferritin, Retinol Binding Protein, (RBP) soluble Tranferrin Receptor (STR).

[1] Department of Human Nutrition, Rolighedsvej 25, 1958 Frederiksberg C

- 1 drop of dry blood on filter paper will be sent to Canada[2] for analysis of essential fatty acid status.

As a precaution, remaining serum will be collected in a tube and will be sent and stored at the University of Copenhagen at -80 degrees C for 5 years in anonymous form. Remaining plasma will be used only to measure other indicators of nutritional status (e.g. Hepcidin), growth and infection related to this study. After 5 years the samples will be destroyed. No analyzes other than the ones mentioned here will be performed.

Thymic Size

The size of the thymus will be measured by ultrasound in a subgroup of children at baseline, at 3 months and 6 months.. The Ultrasound device used will be MicroMaxx model from Sonosite, which is portable, durable and resistant to water and dust. It has a long-lived battery and can thus work in a setting with irregular power supply. The measurement is done with the child lying on the back on a pillow or on mothers lap. The transducer with water-based gel on is placed on the chest of the child just over the sternum, and two pictures in two different angles are obtained. Depending on the mood of the child, the whole examination may take 5- 15 minutes.

To assess the effect of moderate malnutrition on thymic size, a reference group of approximately 30 apparently healthy well-nourished children will be invited to participate..The reference group will be recruited among healthy children attending the health centres for vaccinations or as companions to sick siblings. They will be age-matched to the study participants, and for this reason, they will be recruited by the end of recruitment for the main study, so that the age distribution of the participants is known. Otherwise, the inclusion criteria for these children are

- W/H > -1 z-score
- age 6 – 23 months
- no apparent acute or chronic disease

The data that will be collected from these children is the following

- anthropometry
- morbidity questionnaire
- thymus size measured by ultrasound

---

[1] Dr Juergen Erhardt. Kastanienweg 5, 77731 Willstaett, Germany
[2] Ken Stark, Ph.D., Associate Professor, Department of Kinesiology, University of Waterloo, Waterloo, ON, Canada N2L 3G1

A separate informed consent form will be made for the healthy controls describing the purpose of the study, the assessments that will be carried out and the risks and benefits associated with these. It will be clearly stated that participation is voluntary and that refusal to participate will not affect the treatment that the child is otherwise receiving.

The consent forms will be translated into the local language and read out to childs caretaker. Each participant will receive a copy of the information sheet and consent form. After giving appropriate time to consider, the caretaker will be asked if his/her child may participate in the reference group, and in case they agree, they will be asked to sign the informed consent form. If the caretaker is illiterate, a thumbprint will be accepted instead of a signature, and in this case, a literate witness is required to sign to confirm that the information material has been read out accurately to the caretaker.

No payment will be given to the caretaker for letting his/her child participate in the study's reference group, however, an incentive (a mosquito-net, or something of similar value) will be given. If any condition requiring treatment is identified during the assessments, the child will be referred for further investigation and treatment.

.

3.1.12. Data management

Data will be kept confidential and in a locked facility to which only the investigating study team will have access. All study forms and questionnaires will only be accessible to the study staff during the trial. After the trial completion, these documents will be transferred to University of Copenhagen and kept in a locked area for at least 5 years from the last patient. A copy of the data set will be kept at the study site, and will be managed by ALIMA.

Data will be anonymous. Individuals will receive a unique identification number and no names will be entered into a computer.

Data will be doubled entered into Epidata software by a locally recruited data management team supervised by the Study team. A data management plan will be ensured by the University of Copenhagen. Data will be entered on computers specifically dedicated to this task. Daily back-ups will be made on an external device (USB key or hard drive) dedicated to this task. Data will be sent by email regularly to the principal investigator and co-principal investigators in Europe. Data sent by email will not allow identification of involved subjects.

3.1.13. Data analysis

The main effectiveness criterion will be the mean change in lean body mass after 3 months of supplementation (absolute increase expressed in kg). The primary analysis will be conducted in the intention to treat (ITT) population and will therefore include all randomized subjects having at least one exploitable baseline and 3 months LBM measures,. Theprimary analysis will be conducted by means of a single statistical analysis including both main arms, ie LNS and iCSB:

- o A three-way analysis of variance (ANOVA) will be used to evaluate if the two interventions modify the effect of dehulled soy and soy isolate at different levels of milk content.
- o The ANOVA will also include LBM at baseline as well as a number of additional explanatory (age, sex, MUAC, WHZ, season) that adjust for potential differences present in the study population. To adjust for variation between centers random effects for each centre are also included in the model. Thus, the statistical model will be based on a linear mixed model.
- o Pairwise comparisons of means within and across the two arms will be done using the linear mixed model. Multiplicity will be taken into account by adjusting all pairwise comparisons.
- o The effect of supplementation, ie. the difference in mean changes in LBM at 3 months of each group will be presented in terms of estimated means with 95% confidence intervals.

All other statistical analyses of the primary endpoint, ie other study populations, sub-group analysis, and inside-the-table comparisons, and all statistical analyses of secondary endpoints, will be considered secondary analyses. These include:

- o Evaluation of the effect of the type of soy by comparing, in the LNS and the iCSBs groups separately, the mean increase in LBM at 3 months over the two groups of soy quality (dehulled soy i.e. 3 groups merged, soy isolate i.e. 3 groups merged).
- o Evaluation of the effect of the milk protein content by comparing, in the LNS and the iCSBs groups separately, the mean increase in LBM at 3 months over the three groups of milk protein content (i.e. 2 groups merged for each level of milk).
- o Analysis might also be conducted among the subgroup of participants presenting these 2 LBM measurements and the per-protocol population (i.e. excluding participants with violation of protocol).

For all secondary analyses, no adjustment for multiplicity is planned.

Interim analysis

Due to the relatively short recruitment period (approximately 6 months), no interim analysis will be conducted.

Safety analysis

Safety analyses will be performed among participants having been randomized. The number of adverse events and serious adverse events will be presented for each group. Proportion of participants presenting with AE or serious AE will be compared between groups. Description of adverse events will be given.

Statistical analysis will be performed using Stata software.

3.1.14. Strengths and Limitations to the study

- Randomization should ensure that the effect estimates are not biased by known or unknown confounders.
- HIV status might be an important confounding or modifying factor, but HIV status will not be assessed among the participants. The very low expected HIV sero-prevalence in young children in Burkina Faso should not have substantial consequence on the results of the study.
- Selection biases will be kept to a minimum by ensuring allocation concealment. However, rate of withdrawals (including refusals after randomization) and/or loss to follow-up might differ according to the allocated group. High follow-up and defaulters tracing will be ensured in all arms and incentives will be provided to increase the motivation of all participants.
- Observer biases will be kept to a minimum by blinding the staff responsible for the outcome assessments with respect to the allocated group. In addition, all participants and staff involved in the study will be blind to the milk and soy compositions of the iCSBs and LNS supplements.
- The tracer dilution technique might be difficult to conduct in young children. The study staff will receive an appropriate training and benefit from continuous supervision.
- Possible lack of adherence and contamination between groups may be an important limitation of this study. This study will examine the effectiveness of the food supplementation (rather than the efficacy), that is, a combination of biological/nutritional and logistical/practical effects, which is relevant in practice. Hence, childcare practices related to supplementary feeding and the adherence to the food will be assessed (see

section 3.3). The importance of following the nutritional prescription will be stressed during the health and nutrition education provided to all participants.

- A potential limitation of the study is generalizability, as nutritional status is likely to differ between populations.

3.2. Child care practices related to supplementary feeding and the perception, acceptability of and adherence to the food

Childcare practices related to supplementary feeding and perceptions, acceptability of and adherence to the foods will be explored by doing structured observational studies, in-depth and semi-structured interviews, questionnaires as well as Focus Group Discussions (FGD).

Structured Observations are included in order to gain a realistic picture of actual practices rather than just the informants' views on "correct" perceptions. In this way, this part of the study aims at exploring social activities and the meaning given to these by observing phenomena in their natural setting on a limited subsample.

A trained local assistant will be observing the caretakers and children in the private home of the family during daytime to explore child care practices related to feeding and adherence to treatment and will include observations on particularly:

- *Caregiver's feeding behaviors* such as frequency of and time devoted for supplementary feeding of the child, interaction with the child during feeding incl. level of supervision and encouragement and hygiene behaviors related to the feeding situation(Dearden et al. 2009; Flax et al. 2010)
- *Child feeding practices*, such as the position of the child during the feeding situation, the way the child is fed and utensils used for feeding and how and when the supplementary diets are consumed (i.e. as snacks or as a meal, as individual diets or shared with other household members (Pelto et al. 2003; Flax et al. 2010; Shankar et al. 1998)[i]
- *Adherence to the treatment* in terms of compliance to treatment and to which degree the products are stored, prepared and distributed according to recommendations.

It is planned for the local assistant to spend three consecutive days (during daytime) in the household of each of the participants. For practical reasons, 20 participants will be randomly selected from one of the recruitment sites (10 participants from the LNS groups, 10 participants from the CSB groups)

Furthermore it is planned to carry out 10 semi-structured interviews and 10 focus group discussions (4-6 participants in each). Caretakers will be randomly selected at the recruitment sites but will consist equally of participants receiving either LNS or CSB products. The interviews and FGDs will be carried out at the recruitment site, in a secluded and quiet area, during the waiting time of the visit at three months. The aim of this part of the study will be to explore perceptions about nutrition, malnutrition,the food products, the treatment as well as the acceptability and perceived benefits and/or undesirable effects of the products. A trained research assistant will conduct/moderate the interview/discussions in local language in a place ensuring privacy. The interview/discussions will be audio recorded for later transcription and translation.

A separate informed consent form will be made for participants agreeing to participate in this part of the study describing the purpose of the study. It will be clearly stated that participation is voluntary and that refusal to participate will not affect the treatment that the child is otherwise receiving or the participation in the main study.

The consent forms will be translated into the local language and read out to the child's caretaker. Each participant will receive a copy of the information sheet and consent form. After giving appropriate time to consider, the caretaker will be asked if he/she wishes to participate in the study and allow the research assistant to visit the household for three consecutive days. If they agree, they will be asked to sign the informed consent form. If the caretaker is illiterate, a thumbprint will be accepted instead of a signature, and in this case, a literate witness is required to sign to confirm that the information material has been read out accurately to the caretaker.

Questionnaires: At the first follow-up visit (2 weeks into supplementation) all caretakers of participants in the study will be asked questions on the acceptability of and adherence to the products, following a structured questionnaire. .This questionnaire will include questions on the perception of the quantity, appearance, taste, smell and consistency of the products and will be assessed according to a five-point Hedonic scale (from “5” – “like very much” to “1” – “dislike very much”). Furthermore questions regarding the health of the child and breastfeeding patterns during supplementation as well as feeding practices will be asked.
With the assumption that acceptability of new foods increases, if it is served for a longer period of time, the questionnaire will be repeated at the end of the supplementation period (at 3 months). Furthermore caretakers will at this point be asked questions related to food security based on the assumption that foods may be more acceptable in households with high food insecurity. Food security will be assessed using questions from the Household

Food Security Access Scale (HFIAS)[1], and the level of food insecurity for each household will be classified into four levels of food insecurity (food secure, mildly food insecure, moderately food insecure, severely food insecure) (Annex 8). Furthermore a trained research assistant will administer the questionnaire in the local language.

4. Ethical and administrative considerations

4.1. Authorizations

The study protocol has been granted approval from the Ethical committee of Burkina Faso (August 2012, see annex 9) andthe Danish National Committee on Biomedical Research Ethics (September 2012, see annex 10). Furthermore the study has recieved authorisation to import and export study related material from the Direction Génerale de la Pharmacie du Médicament et des Laboratoires (DGPML) under the Ministry of Health in Burkina Faso. The study will be conducted in accordance with the principles of the Declaration of Helsinki and international ethical guidelines for biomedical research involving human subjects, published by the Council for International Organizations of Medical Sciences (CIOMS). Prior to the study, community leaders will be informed about the purpose of the study. Authorization for the study will be obtained from the Ministry of Health, Burkina Faso.

4.2. Informed Consent

The consent process will include separate information notices and consent forms for the trial, and for the “practices, perception, acceptability, and adherence” study, (annexes 11, 12 and 13). All documents will be translated into local languages, and back-translated to ensure accuracy. For all children eligible for participation in the trial, the trial information notice will be explained orally to their parents or guardians who will receive a printed copy. After answering any questions about the study, the investigator will ask the parent or caretaker, keen to participate, to sign (or fingerprint if the signature can’t be obtained) the trial consent form. Any activities related to the study other than the pre-screening (W/H and MUAC) will only be done after the caretaker or parent has signed the informed consent. Copies of the written informed consent will be given to the parent or caretaker and another copy will be kept at of the office of ALIMA in Yako. A literate witness will be required to be present during the reading of the consent form and sign in addition to the fingerprint requested from a non-

[1] Coates J, Swindale A & Bilinsky P (2007)Household Food Insecurity Access Scale (HFIAS) for Measurement of Food Access:Indicator Guide. Food and Nutrition Technical Assistance Project (FANTA)

literate study participant. The same procedure will be followed for the caretakers invited to participate in the pilot study or the “practices, perception, acceptability, and adherence” study, using the appropriate information notice and consent form.

Any personal data (case reports, laboratory records, registers, or other forms, audio and video records) will be kept confidential and will not be disclosed to anyone outside the study teams. The electronic database will not contain names or addresses of patients to ensure their anonymity.

All children recruited in the study in need of medical treatment, will be treated according to National Guidelines. The inclusions in the study will be made voluntarily and refusals to participate in the study will have no influence on the medical treatment provided to the child in the health center. Refusing to participate in the pilot study will not have any influence on the participation in the trial.

4.3. Risks and benefits

There is no major risk associated to this research project.

There are no obvious risks related to the tracer dilution test and no danger related to the deuterium solution. The tracer deuterium is a stable, naturally-occurring isotope. Stable isotopes have been used in human metabolic studies for more than fifty years. Tissue concentrations below 15% have not been associated with harmful effects. Trivial transitory side effects as transitory episodes of vertigo have been reported at concentrations around 0.35-0.65%. However, the amount of tracer consumed in studies of body composition such as this, enrich body water to a maximum of 0.1%. At this level no adverse side effects have been reported (IAEA). However, discomfort may occur during saliva sampling, especially for the youngest children. This procedure requires concentration and focus of the participant, which may be difficult for a small child to uphold. All measures will be made to reduce discomfort as much as possible.

Participants in this study might suffer discomfort related to the blood sampling. To reduce discomfort, an analgesic ointment will be applied to the skin of the child prior to blood sampling. Blood collection may cause localized pains, small hematoma following insertion of the needle, or, rarely, phlebitis. A maximum of 2x2ml of blood will be collected. The amount of blood removed will be too small to affect the child's health. Blood sampling will be attempted in two tries for each child. If not successful, the procedure will be postponed to the next follow-up visit.

Finally, allergic reactions such as rash, vomiting/diarrhoea, respiratory problems or allergic shock within the first hour of eating the food are a potential risk. The first dose of the

products will therefore be eaten under supervision of the study staff and in case of allergic reactions, relevant measures will be taken.

The study will include various benefits for the participants: food distribution, incentives, and nutritional and health education. In addition, each child will benefit from a fortnightly or monthly close clinical follow-up and, if found sick (or severely malnourished), would receive standard therapy following national clinical guidelines.

Finally, the results will help adapt the guidelines for the treatment of MAM in Burkina Faso and will have an essential programmatic effect on the future of ALIMA's nutritional program strategy. If acceptable and cost-effective products were identified for the management of MAM, this would furthermore benefit communities prone to food insecurity and high malnutrition rates.

In addition, this study will also strengthen local research capacities through the training of local research staff members and technicians.

4.4. Food producers

The food products will be produced by GC Rieber Compact, a private Norwegian company created in 1948 and with extensive experience in among other things developing and producing nutritional products for the prevention and treatment of malnutrition. The nutritional supplementation products has been developed in collaboration with the University of Copenhagen and technical advisors from WFP and GC Rieber Compact based on the position paper from WHO on the composition of foods for children with MAM. The 12 food supplements all contain the same micronutrient composition but differ in the amount of fat, protein, quality of soy and percentage of protein provided by DSM. A detailed description of the composition of the different products can be found in annex 2.

The shelf life of iCSBs and LNS is 9 and 24 months respectively. Shipment of food supplements to the project site will take shelf life into account.

GC Rieber Compact has not been involved in the design of the study, nor will they be involved in the analysis and interpretation of the data.

A supply agreement has been made between GC Rieber Compact, MSF Denmark and University of Copenhagen for the development, production and future use of the nutritional supplementation products developed for this study. This agreement ensures that results of this study are placed in the public domain, meaning that any producer, not only GC Rieber Compact, are free to produce the final products.

4.5. Commitment of ALIMA to use the results of the trial and sustainability of the intervention

A the end of the trial, ALIMA is willing to use the most relevant strategy for the management of MAM identified through the trial as part of its nutritional activities in Burkina Faso and other countries. The results of this study would be communicated to and benefit the beneficiaries of UN agencies and NGOs involved in the management of childhood acute malnutrition.

4.6. Collaborative partnership

The trial's sponsor is Department of Nutrition, Sports and Exercise, University of Copenhagen in a research collaboration with theMédecins sans Frontières Denmark. This proposal was developed following advice of technical referents on trial design, conduct, and analysis, composed of leading experts in nutrition. All investigators in this study are financially independent from this study and no conflicts of interest are reported.

It is a strong wish to involve researchers from the Institut de Recherche en Science de la Santé, Burkina Faso well as to collaborate with the nutrition department of the Ministry of Health. The exact nature of the collaboration with the IRSS is currently being elaborated. So far staff from the IRSS have been involved in the project one of which will be supported to do a PhD. as

4.7. Financing and Insurance

The trial is funded by Danida and MSF-Norway. Insurance has been taken with HDI-Gerling Industrie Versicherung which is a Europe-based accredited company. The insurance covers 1848 children (including children recruited for the pilot) and covers indemnification against all sums that the Study sponsor should become legally liable to pay, namely damages resulting from bodily injury arising from the Study up to 2 years after completion of the Study. See separate insurance policy in annex 14.

4.8 Communications

The study results will be communicated in Burkina Faso (written report and oral presentation) to the health authorities of Burkina Faso and to the community of Province de Passoré, involved in the study.

Publications will be written with the input of all co-investigators and supervisory staff and submitted to international peer-reviewed journals.

5. Study schedule and training

. Training of the field team will be ensured by ALIMA and the PhD students of the University of Copenhagen involved in the project. In addition, all partners will ensure regular support. During the study, continuous supervision, regular meetings and reporting will ensure updating of knowledge and permanent training throughout the study.

Recruitment of the children is expected to begin in August 2013 and last for 6 months (Table 3). For each child, the intervention is scheduled for three months, with an additional three months of follow-up of all children participating in the trial. In total, the study is expected to last for one year including follow-up period.

Table 3 Study schedule

Year		2013												2014								
Month		1	2	3	4	5	6	7	8	9	10	11	12	1	2	3	4	5	6	7	8	9
Pre-study	Pilots	X	X																			
	Adjustments after pilots			X	X	X																
Study	Inclusion								X	X	X	X	X	X								
	Supple-mentation								X	X	X	X	X	X	X	X	X					
	Follow-up											X	X	X	X	X	X	X	X	X		

6. References

1. Aaby P, Marx C, Trautner S, Rudaa D, Hasselbalch H, Jensen H, Lisse I.: Thymus size at birth is associated with infant mortality: a community study from Guinea.Bissau. Acta Oaediatr. 2002;91(6):698-703

2. Abebe Y, Stoecker B.J, Hinds M.J, Gates G.E: Nutritive value and sensory acceptability of corn- and kocho based foods supplemented with legumes for infant feeding in Southern Ethiopia. *Ajfand Online,* 2006; 6 (1).

3. Adu-Afarwuah S, Lartey A, Brown KH, et al. Randomized comparison of 3 types of micronutrient supplements for home fortification of complementary foods in Ghana: effects on growth and motor development. *Am J Clin Nutr*. 2007;86(2):412-20.

4. Adu-Afarwuah, S., Lartey, A., Zeilani, M. and Dewey, K. G. , Acceptability of lipid-based nutrient supplements (LNS) among Ghanaian infants and pregnant or lactating women. *Maternal & Child Nutrition,* no. doi: 10.1111/j.1740-8709.2010.00286.x

5. Adu-Afarwah, S., et al. Home fortification of complementary foods with micronutrient supplements is well-accepted and has positive effects on infant iron status in Ghana. *Am J Clin Nutr.* 2008; 87:929 – 38

6. Agostoni C, Axelsson I, Goulet O, et al. Soy protein infant formulae and follow-on formulae: a commentary by the ESPGHAN Committee on Nutrition. *J Pediatr Gastroenterol Nutr*. 2006;42(4):352-61.

7. Andang'o PE, Osendarp SJ, Ayah R, West CE, Mwaniki DL, De Wolf CA, Kraaijenhagen R, Kok FJ, Verhoef H. Efficacy of iron-fortified whole maize flour on iron status of schoolchildren in Kenya: a randomised controlled trial. Lancet. 2007;369(9575):1799-806.

8. Beard JL, Murray-Kolb LE, Rosales FJ, Solomons NW, Angelilli ML. Interpretation of serum ferritin concentrations as indicators of total-body iron stores in survey populations: the role of biomarkers for the acute phase response. Am J Clin Nutr. 2006;84(6):1498-505.

9. Beaton GH, Ghassemi H. Supplementary feeding programs for young children in developing countries. *Am J Clin Nutr*. 1982;35(4 Suppl):863-916.

10. Bentley, M. B et al. Maternal feeding behavior and child acceptance of food during diarrhea episodes, convalescence and health in the Central Northern Sierra of Peru. *American Journal of Public Health*.1991: 83, 1-5.

11. Black RE, Allen LH, Bhutta ZA, et al. Maternal and child undernutrition: global and regional exposures and health consequences. *Lancet*. 2008;371(9608):243-60.

12. Briend A, Dewey KG, Reinhardt GA. Fatty acid status in early life in low-income countries- overview of the situation, policy and research priorities. *Maternal and Child Nutrition.* 2011; 7 (Suppl. 2), pp. 141–148

13. Chaparro CM, Dewey KG. Use of lipid-based nutrient supplements (LNS) to improve the nutrient adequacy of general food distribution rations for vulnerable sub-groups in emergency settings. *Maternal and Child Nutrition.* 2010; 6 (Suppl. 1): 1–69.

14. Ciliberto MA, Sandige H, Ndekha MJ, et al. Comparison of home-based therapy with ready-to-use therapeutic food with standard therapy in the treatment of malnourished Malawian children: a controlled, clinical effectiveness trial. *Am J Clin Nutr* 2005;81:864–70.

15. Cliff DP, Reilly JJ, Okely AD. Methodological considerations in using accelerometers to assess habitual physical activity in children aged 0-5 years. *J Sci Med Sport.* 2009;12(5):557-67. Epub 2009 Jan 14.

16. De Pee S, Bloem MW. Current and potential role of specially formulated foods and food supplements for preventing malnutrition among 6-23 months old and treating moderate malnutrition among 6-59 months old children. *Food Nutr Bull.* 2009;30(3 Suppl):S434-63.

17. De Vries S I, Bakker I, Hopman-Rock M, Hirasing RA, van Mechelen W: Clinimetric review of motion sensors in children and adolescents. *J Clin Epidemiol* 2006; 59: 670-680.

18. Decsi T, Molnár D, Koletzko B. The effect of under- and overnutrition on essential fatty acid metabolism in 37. childhood. *Eur J Clin Nutr*. 1998;52(8):541-8.

19. Defourny I, Seroux G, Abdelkader I, Harczi G. Management of moderate acute malnutrition with RUTF in Niger. *ENN Field exchange*, 2007;31:2-4. Available at: http://www.ennonline.net/fex/31/fex31.pdf [Accessed February 21, 2009].

20. Doherty CP, Crofton PM, Sarkar MAK, Shakur MS, Wade JC, Kelnar CJH, Elmlinger MW, Ranke MB, Cutting WA. Malnutrition, zinc supplementation and catch-up growth: changes in insulin-like growth factor I, its binding proteins, bone formation and collagen turnover. *Clinical Endocrinology* 2002; 57:391–399.

21. Diouf A et al. *Validity of impedance-based predictions of total body water as measured by 2H dilution in African HIV/AIDS outpatients. Br J Nutr*. 2009 May ; 101(9): 1369–1377. doi:10.1017/S0007114508067640.

22. Dioum A et al. *Validity of impedance-based equations for the prediction of total body water as measured by deuterium dilution in African women. Am J Clin Nutr* 2005;81:597– 604.

23. Erdman JW, Badger TM, Lampe JW, Setchell KDR, Messina M. Not all soy products are created equal: caution needed in interpretation of research results. *J Nutr*. 2004;134(5):1229S-1233S.

24. Filteau SM, Morris SS, Abbott RA, et al. Influence of morbidity on serum retinol of children in a community-based study in northern Ghana. *Am J Clin Nutr*. 1993;58(2):192-7.

25. Flax VL et al. Malawian mother's attitudes towards the use of two supplementary foods for moderately malnourished children. *Appetite.* 2009; 53, 195-202.

26. Fomon SJ, Haschke F, Ziegler EE, Nelson SE. Body composition of reference children from birth to age 10 years. *Am J Clin Nutr*. 1982;35(5 Suppl):1169-75.

27. Fratesi JA, Hogg RC, Young-Newton GS, Patterson AC, Charkhzarin P, Block Thomas K, Sharrat MT, Stark KD. Direct quantitaion of omega-3 fatty acid intake in Canadian residents of a long-term care facility. Appl. Physiol. Nutr. Metab. 34:1-9. 2009.

28. Freidlin B, Korn EL, Gray R, Martin A. Multi-arm clinical trials of new agents: some design considerations. *Clin Cancer Res*. 2008;15;14(14):4368-71.

29. Garly ML, Trautner SL, Marx C, Danebod K, Nielsen J, Ravn H, Martins CL, Balé C, Aaby P, Lisse IM: Thymus size at 6 months of age and subsequent child mortality. J Pediatr. 2008 Nov; 153(5):683-8

30. Gladstone M.,Lancaster GA, Umar E, Nyirenda M, Kayira E, van den Broek NR, Smyth RL: The Malawi Development Assessment Tool (MDAT): The creation, Validation and Reliablility of a Tool to Assess Child Development in Rural African Settings. *PLoS Med*. 2010 May 25;7(5):e1000273

31. Golden M. Proposed nutrient requirements of moderately malnourished populations of children. In: Vol 2008. Geneva: WHO.

32. Halton TL, Hu FB. The effects of high protein diets on thermogenesis, satiety and weight loss: a critical review. J Am Coll Nutr. 2004;23(5):373-85.

33. Hess, S. Y., Bado, L., Aaron, G. J., Ouédraogo, J.-B., Zeilani, M. and Brown, K. H. , Acceptability of zinc-fortified, lipid-based nutrient supplements (LNS) prepared for young children in Burkina Faso. *Maternal & Child Nutrition,* no. doi: 10.1111/j.1740-8709.2010.00287.x

34. Hoppe C, Andersen GS, Jacobsen S, et al. The use of whey or skimmed milk powder in fortified blended foods for vulnerable groups. *J Nutr*. 2008;138(1):145S-161S.

35. International Atomic Energy Agency (IAEA). Introduction to body composition assessment using the deuterium dilution technique with analysis of saliva samples by Fourier TransformInfrared Spectrometry (FTIR). Forthcoming Human Health Series (In press).

36. Isanaka S, Nombela N, Djibo A, et al. Effect of preventive supplementation with ready-to-use therapeutic food on the nutritional status, mortality, and morbidity of children aged 6 to 60 months in Niger: a cluster randomized trial. *JAMA*. 2009;301(3):277-85.

37. Jeppesen DL: The size of the thymus: an important immunological diagnostic tool? Acta Paediatr. Sept 2003:92: 994-6

38. Kouanda S, Doulougou B, De Coninck V, Habimana L, Sondo B, Tonglet R, Ketelslegers JM, Robert A. Insulin Growth Factor-I in Protein-Energy Malnutrition during Rehabilitation in Two Nutritional Rehabilitation Centres in Burkina Faso. *J Trop Med*. 2009;2009:832589. Epub 2009 Mar 17.

39. Kuusipalo H, Maleta K, Briend A, Manary M, Ashorn P.Growth and change in blood haemoglobin concentration among underweight Malawian infants receiving fortified spreads for 12 weeks: a preliminary trial. J *Pediatr Gastroenterol Nutr*. 2006;43(4):525-32.

40. LaGrone L, Trehan I, Meuli GJ, Wang RJ, Thakwalakwa C, Maleta K, Manary MJ. A novel fortified blended flour, corn-soy blend “plus-plus,” is not inferior to lipid-based ready-to-use supplementary foods for the treatment of moderate acute malnutrition in Malawian children. Am J Clin Nutr 2012;95:212–9.

41. Laine M, Jemmy JP, Delchevalerie P. Enquête nutritionnelle et de mortalité rétrospective, district sanitaire de Guidan Roumdji, Région de Maradi, Niger. Novembre 2010.

42. Lopriore C, Guidoum Y, Briend A, Branca F. Spread fortified with vitamins and minerals induces catch-up growth and eradicates severe anemia in stunted refugee children aged 3-6 y. *Am J Clin Nutr.* 2004;80(4):973-81.

43. Manary MJ, Sandige HL. Management of acute moderate and severe childhood malnutrition. *BMJ*. 2008;337:a2180.

44. Matilsky DK, Maleta K, Castleman T, et al. Supplementary feeding with fortified spreads results in higher recovery rates than with a corn/soy blend in moderately wasted children. J *Nutr 2009;*139:773–8.

45. McLaren DS. The great protein fiasco. *Lancet*. 1974;2(7872):93-6.

46. Michaelsen KF, Hoppe C, Roos N, et al. Choice of foods and ingredients to give to moderately malnourished children 6 months to 5 years olds. Geneva: WHO/UNICEF/WFP/UNHCR technical consultation. *Food and Nutrition Bulletin*, 2009, in press.

47. Michaelsen KF, Skov L, Badsberg JH, Jørgensen M. Short-term measurement of linear growth in preterm infants: validation of a hand-held knemometer. *Pediatr Res.* 1991;30(5):464-8.

Michaelsen KF. Short-term measurements of linear growth in early life: infant knemometry. *Acta Pediatr* 1997; 86:551 -3.

48. Ministere de la Santé, Direction de la Nutrition Burkina Faso. Rapport d’enquete nutritionelle nationale. 2011.

49. Ministère de la Santé, District Sanitaire de Yako. Plan d’action 2012. 2011

50. Nackers F, Broillet F, Oumarou D, Djibo A, Gaboulaud V, Guerin PJ, Rusch B, Grais RF, Captier V. Effectiveness of ready-to-use therapeutic food compared to a corn/soy-blend-based pre-mix for the treatment of childhood moderate acute malnutrition in Niger. *J Trop Pediatr.* 2010 Mar 23.

51. Navarro-Colorado C. Retrospective Study of Emergency Supplementary Feeding Programmes June 2007. Available at: http://www.ennonline.net/pool/files/research/Retrospective_Study_of_Emergency_Supplementary_Feeding_Programmes_June%202007.pdf [Accessed February 21, 2009].

52. Patel MP, Sandige HL, Ndekha MJ, et al. Supplemental feeding with ready-to-use therapeutic food in Malawian children at risk of malnutrition. *J Health Popul Nutr* 2005;23:351–7.

53. Praygod G, Range N, Faurhold-Jepsen D, Jeremiah K, Faurhold-Jepsen M, Aabye MG, Magnussen P, Changaluch J, Andersen AB, Wells JC and Friis H. Sex, smoking and socioeconomic Status are associated with body composition among tuberculosis patients in a deuterium dilution cross-sectional study in Mwanza, Tanzania. J Nut 2013 May; 143(5):735-41

54. Prentice AM. Macronutrients as sources of food energy. *Public Health Nutr* 2005;8:932-9.

55. Phuka JC, Maleta K, Thakwalakwa C, Cheung YB, Briend A, Manary MJ, Ashorn. Complementary feeding with fortified spread and incidence of severe stunting in 6- to 18-month-old rural Malawians. *P. Arch Pediatr Adolesc Med.* 2008 Jul;162(7):619-26.

56. Phuka JC, Maleta K, Thakwalakwa C, Cheung YB, Briend A, Manary MJ, Ashorn P. Postintervention growth of Malawian children who received 12-mo dietary complementation with a lipid-based nutrient supplement or maize-soy flour. *Am J Clin Nutr.* 2009;89(1):382-90.

57. Reichert FF, Baptista-Menez AM, Wells JC, Carvalho Dumith S, Hallal PC: Physical activity as a predictor of adolescent body fatness: a systematic review. Sports. Med.; 2009;39(4):279-94

58. Rocquelin G, Tapsoba S, Kiffer J, Eymard-Duvernay S. Human milk fatty acids and growth of infants in Brazzaville (The Congo) and Ouagadougou (Burkina Faso). *Public Health Nutr*. 2003;6(3):241-8.

59. Roubenoff R, Kehayias. The meaning and measurement of lean body mass. *JJ.Nutr*. Rev. 1991; 49(6):163-75.

60. Rowe J.P, Brodegard W.C, Pike O.A, Steele, F.M, Dunn, M.L: Storage, preparation and usage of fortified food aid among Guatemalan, Ugandan and Malawian beneficiaries; A field study report. Food Nutr Bull. 2008;29(3):213-20.

61. Santon, I.S. et al. Evaluation of the impact of a nutritional program for undernourished children in Brazil. *Cadernos de Saude Publica*. 21, 776-785. 2005

62. Savino W, Dardenne M: Nutritional imbalances and infections affect the thymus: consequences on T-cell-mediated immune responses. Proc. Nutr. Soc. 2010 Nov;69(4):636-43

63. Sauerwein HP, Serlie MJ. Optimal nutrition and its potential effect on survival in critically ill patients. *Neth J Med.* 2010 68(3):119-22.

64. Shankar, A. V et al. Eating from a Shared Plate Affects Food Consumption in Vitamin A–Deficient Nepali Children. American Society for Nutritional Sciences. 1998

65. Squali Houssaïni FZ, Foulon T, Payen N, Iraqi MR, Arnaud J, Groslambert P. Plasma fatty acid status in Moroccan children: increased lipid peroxidation and impaired polyunsaturated fatty acid metabolism in protein-calorie malnutrition. *Biomed Pharmacother*. 2001;55(3):155-62.

66. Suarez FL, Springfield J, Furne JK, et al. Gas production in human ingesting a soybean flour derived from beans naturally low in oligosaccharides. *Am J Clin Nutr*. 1999;69(1):135-9.

67. Thakwalakwa C, Ashorn P, Phuka J, Cheung YB, Briend A, Puumalainen T, Maleta K. A lipid-based nutrient supplement but not corn-soy blend modestly increases weight gain among 6- to 18-month-old moderately underweight children in rural Malawi. *J Nutr.* 2010; 140(11):2008-13.

68. The Sphere Project, Humanitarian Charter and Minimum Standards in Disaster Response: New Sphere Standards for Food Security, Nutrition and Food Aid, available on;

http://www.sphereproject.org/component/option,com_docman/task,cat_view/gid,17/Itemid,203/lang,english/

69. Uauy R, Castillo C. Lipid requirements of infants: implications for nutrient composition of fortified complementary foods. J Nutr. 2003;133(9):2962S-72S.

70. UNDP. Human development report 2011. [Available at: http://hdr.undp.org/en/statistics/hdi/]

71. UNICEF. Strategy for improved nutrition of children and women in developing countries. New York: UNICEF 1990

72. Victoria CG, Adair L, Fall C, Hallal PC, Martorell R, Richter L, Sachdev H. Maternal and child undernutrition: consequenses for adult health and human capital. *The Lancet, 2008;*371:340-357

73. Webb, P., B. Rogers, I. Rosenberg, N. Schlossman, C. Wanke, J. Bagriansky, K. Sadler, Q. Johnson, J. Tilahun, A. Reese Masterson, A. Narayan. 2011. Delivering Improved Nutrition: Recommendations for Changes to U.S. Food Aid Products and Programs. Boston, MA: Tufts University.

74. Wells JC, Fewtrell MS. Is body composition important for paediatricians? *Arch Dis Child.* 2008;93(2):168-72.

75. Wells JC, Hawton K, Darch T, Lunn PG. Body composition by 2H dilution in Gambian infants: comparison with UK infants and evaluation of simple prediction methods. *Br J Nutr.* 2009;102(12):1776-82.

76. Wells JCK, Fewtrell MS. Measuring body composition. Arch Dis Child. 2006; 91(7): 612–617.WFP. Improving corn soy blend and other fortified blended foods. Why and how? *Sight and Life*, 2008;1:26-31. Available at: http://www.sightandlife.org/images/stories/pageimages/content/magazine/03_2008/supplement_10_minutes_to_learn.pdf [Accessed February 21, 2009].

77. WFP. Improving corn soy blend and other fortified blended foods. Why and how? Sight and Life, 2008;1:26-31. Available at: http://www.sightandlife.org/images/stories/pageimages/content/magazine/03_2008/supplement_10_minutes_to_learn.pdf [Accessed February 21, 2009].

78. WHO Multicentre Growth Reference Study Group: Motor Development Study: Windows of achievement for six gross motor development milestones. *Acta Pædiatrica,* 2006; Suppl 450: 86-95

79. World Health Organization. WHO child growth standards: head circumference-for-age, arm circumference-forage, triceps skinfold-for-age and subscapular skinfold-for-age: methods and development. Geneva; 2007.

80. WHO. Technical Note: Supplementary foods for the management of moderate acute malnutrition in infants and children 6 to 59 months of age. WHO: 2012.[available from: http://apps.who.int/iris/bitstream/10665/75836/1/9789241504423_eng.pdf]

Summary of changes to the protocol:

A protocol, version 50, had received ethical approval from the ethics committee in Burkina Faso in November 2012. Upon approval, a pilot study was carried out January-March 2013. Following completion of the pilot, minor technical changes in methods were introduced in the protocol such as a fixed three hours equilibration time for the diluted deuterium technique. Also, there was a change of scientific sponsor. Therefore, version 52 attached above is therefore the original, as well as the final approved, protocol sent to Ethics Committee with no subsequent amendments.

Summary of changes to the statistical analysis plan:

Compared to the statistical plan outlined in the protocol, we eventually decided to deviate on two issues:

1) Our primary outcome was fat-free mass accretion over three months, adjusted for length. We have not changed the primary outcome, but decided to do the length adjustment by dividing with length in meters squared, ie expressing fat-free mass accretion as an index. There are several reasons for this: First, we found that dividing by length in meters squared was the optimal adjustment. Second, since children are growing in length, then it is not enough to adjust for baseline length. Using fat-free mass index at both time points enable us to adjust for not only baseline length, but also changes in length over time.

2) We had initially planned to adjust for multiplicity, in case we had to compare 12 groups. However, while this reduces the risk of type 1 errors, it also increases the risk of type 2 errors. We therefore eventually followed standard practice of not adjusting p-values for reduced factorial model. In other words, for the pairwise comparisons within factors in the reduced models no multiplicity adjustments were applied.
